# The effects of neighbourhood social cohesion on preventing depression and anxiety among adolescents and young adults: rapid review

**DOI:** 10.1192/bjo.2022.57

**Published:** 2022-06-01

**Authors:** Josefien J. F. Breedvelt, Henning Tiemeier, Evelyn Sharples, Sandro Galea, Claire Niedzwiedz, Iris Elliott, Claudi L. Bockting

**Affiliations:** Academic Medical Center, Amsterdam University Medical Centers, University of Amsterdam, The Netherlands; Centre for Urban Mental Health, University of Amsterdam, The Netherlands; and National Centre for Social Research, UK; Maternal and Child Center of Excellence, Harvard T.H. Chan School of Public Health, Massachusetts, USA; Independent Consultant, Barnardo's, UK; Boston University School of Public Health, Massachusetts, USA; Institute of Health and Wellbeing, University of Glasgow, UK; Department of Policy and Research, Irish Human Rights & Equality Commission, Ireland; Academic Medical Center, Amsterdam University Medical Centers, University of Amsterdam, The Netherlands; and Centre for Urban Mental Health, University of Amsterdam, The Netherlands

**Keywords:** Common mental health conditions, community prevention, public health, neighbourhoods

## Abstract

**Background:**

Research suggests that increasing neighbourhood social cohesion can prevent mental health problems, including depression and anxiety. However, it is unknown whether this is the case for adolescents and young adults.

**Aims:**

To investigate whether neighbourhood social cohesion can prevent depression and anxiety, and identify interventions that can increase neighbourhood cohesion in young people.

**Method:**

We conducted a rapid review for an overview of the available literature. PubMed, Campbell Collaboration, KSR Ltd and grey literature databases were searched from inception up to 10 July 2020. When synthesising the results, we applied a hierarchy of evidence, prioritising study designs that allowed for the most ability to infer causality. Risk of bias was assessed with the ROBIS tool and Joanna Briggs Institute risk-of-bias assessment. A narrative review and two workshops with young people were conducted to inform what future interventions may look like.

**Results:**

Forty-two peer-reviewed publications, including two systematic reviews, 13 longitudinal studies and 27 cross-sectional studies, were identified. Prospective longitudinal studies found that neighbourhood social cohesion factors (safety, trust, positive social connections, helping others and a lack of crime and violence) were associated with fewer depressive symptoms. Future interventions to increase neighbourhood cohesion should involve creating safe and attractive community centres, accessible and safe outdoor spaces, community activity groups and online communities.

**Conclusions:**

Neighbourhood social cohesion has the potential to protect mental health. The next step is to conduct intervention studies to evaluate the effects on onset prevention. Clinicians should consider the impact cohesion can have on mental health, and signpost to community initiatives.

## Background

Common mental health conditions are associated with high levels of disability and recurrence worldwide.^1^ Young people are at an increased risk of developing such conditions. Anxiety disorders often first occur during adolescence, and young adulthood is a key risk period for developing depression.^2^ Neighbourhood social cohesion may mitigate the risk of developing depression or anxiety among young people.^3^ Evidence from longitudinal and cross-sectional studies suggests that neighbourhood social cohesion may protect against the adverse mental health effects of growing up in poverty, both for children and young people^4,5^ and adult populations.^6–8^

Neighbourhood social cohesion can be defined by the presence of social cohesion at a neighbourhood level. Social cohesion can be defined as ‘the extent of connectedness and solidarity among groups in society’ and consists of the absence of latent social conflict (e.g. polarisation, racial/ethnic tensions) and the presence of strong social bonds (e.g. trust, reciprocity), social connection and institutions of conflict management.^9^ Neighbourhood social cohesion is operationalised at the level of a neighbourhood or community.^10^ Neighbourhoods can be defined as geographical places that have social and cultural meaning to residents and non-residents alike, and are subdivisions of large places.^11^

Although factors of neighbourhood social cohesion, such as inequality,^12^ racial and ethnic tensions^13^ and a lack of social support,^14^ are known risk factors for mental health problems, a systematic overview of the potential effect of neighbourhood social cohesion on depression and anxiety in young people is lacking. Reviews to date have either been conducted over 6 years ago, with new research warranting an updated review^15^; studied effects of social cohesion or social capital, but did not include a neighbourhood component;^14^ or primarily focused on (older) adults^16,17^ or adolescents.^18^ Moreover, young people's views and priorities have not yet been included in prior reviews on this topic. Although it is still uncommon, including the views of young people with lived experience may help identify implications of results and improve the practical solutions and impact delivered by reviews, by delivering data-driven interpretations from lived experience.^19^ Neighbourhood social cohesion can be a new target for interventions to prevent depression and anxiety. To date, the evidence on the effectiveness of prevention in adolescents and young adults is mixed. Evidence suggests that preventative interventions for adolescents and young adults are effective in reducing depression and anxiety symptoms and onset in the short term;^20,21^ however, no study has examined the sustainability of the effect for over 12 months, and evidence that interventions can prevent the onset of a depressive episode in young adults is lacking.^21^ This supports the need for the identification of new targets for prevention interventions.

A comprehensive review that explores the potential of neighbourhood social cohesion for preventing anxiety and depression in adolescents and young adults is needed. This review will explore the following questions:

- Etiological: Which elements of neighbourhood social cohesion affect the mental health (depression and anxiety) of adolescents and young people (14–24 years), and to what extent?
- Intervention: What is the effectiveness of current interventions aiming to improve neighbourhood social cohesion for preventing depression and/or anxiety in adolescents and young people (14–24 years), and what could future interventions look like?
- Young people's perspectives: What are the perspectives of young people toward the elements of neighbourhood social cohesion and related potential interventions that may affect their mental health?

## Method

A rapid review was conducted. PubMed; Campbell Collaboration; KSR Evidence; OAIster; OpenGrey; Jisc Library Hub Discover; DH-DATA: Health Administration, Medical Toxicology & Environmental Health (Dialog); and Google were searched from inception until 10 July 2020 (for search strings see Supplementary File 1 available at https://doi.org/10.1192/bjo.2022.57). The protocol of this review can be found in Supplementary File 2. Included study designs were randomised controlled trials, quasi-experimental studies, longitudinal cohort studies and cross-sectional studies. Studies were included if participants were aged 14–24 years (based on mean age assessment), the study focused on neighbourhood social cohesion, social capital or community cohesion, and the study outcome was depression and anxiety as measured with a validated questionnaire. Studies were excluded if they focused on adolescents and young adults with a primary diagnosis of a long-term health condition (i.e. diabetes or HIV) or externalising conditions such as addictions or non-disorder specific outcome measures (e.g. well-being, resilience, loneliness, self-esteem; see Supplementary File 3 for a Population, Intervention, Comparison, Outcomes and Study design table). Peer-reviewed articles were screened by J.J.F.B. and E.S.; full-text inclusion decisions were made by J.J.F.B., Edyta Ryczek and E.S. Extractions were conducted by Edyta Ryczek, with a second author randomly checking 20% of extractions. Covidence (Veritas Health Innovation, Australia; see www.covidence.org) was used for importing studies and removal of all duplicates.

Study results were described via a narrative synthesis with priority on describing study results that would allow for causal inference with, in order of priority meta-analyses, randomised controlled trials, quasi-experimental study designs and longitudinal study designs. Cross-sectional studies were only described if no longitudinal study designs were available.

For the second research aim, we prioritised describing results from the rapid review first. However, if there were no studies that met our inclusion criteria, we set out to provide an overview of what potential interventions that could improve neighbourhood social cohesion could look like.

### Risk of bias

Risk of bias in longitudinal studies was assessed with the Joanna Briggs Institute Critical Appraisal Checklist for Case Series.^22^ Risk of bias in the systematic reviews was assessed with the Risk of Bias Assessment Tool for Systematic Reviews (ROBIS).^23^ Cross-sectional studies were assessed with the Joanna Briggs Institute Critical Checklist for Cross-Sectional Studies.^24^

### Lived experience workshops

Two consultative workshops with young people were organised and held by Leaders Unlocked (leaders-unlocked.org), to inform the review. First, a workshop with young people was held to inform the search terms and a theoretical conceptual framework of neighbourhood social cohesion. This was because we identified a broad range of definitions and interpretations of neighbourhood social cohesion. We set out to provide a theoretical conceptual framework to give structure to our results and interpretations. Such a framework could also be a useful basis for future research. A unique feature of our proposed conceptual framework is that it includes the views of young people, whereas most theoretical conceptual frameworks to date have not explicitly included the views of people with lived experience.

The first lived experience workshop explored what neighbourhood social cohesion looked like to them. A qualitative thematic analysis was applied to summarise the key factors of neighbourhood social cohesion. A narrative review of definitions on neighbourhood social cohesion and social capital was conducted alongside the workshop, and provided theoretical definitions of neighbourhood social cohesion. Table 1 provides an overview of the seven identified components of neighbourhood social cohesion, their associated definitions and an illustrative quote from young people as to what this meant to them.

**Table 1:** A conceptual theoretical framework of neighbourhood social cohesion

Factors of neighbourhood social cohesion	Definition of factor	Illustrative quote from workshop
Relationships	Positive relationships with peers in a local area,^25^ which consists of the extent of positive intergroup relationships and the processes that form these,^26^ as well as an abundance of relationships crossing social divides^9^	‘When I was really struggling and walking around saying hi to people and getting a hi back can make your day … ’
Safety	Perceived safety and neighbours facilitating safety.^4^ Includes social order upheld by the absence of violence and crime and acceptance of diversity and tolerance toward minorities^27^Also includes an absence of social conflict (i.e. polarisation, racial/ethnic tensions) and the neighbourhood is free from the (direct) exposure to violence (witness or threat)^9^	‘There isn't any place where you feel like you shouldn't go, you can't go down that alleyway because you're not the right race or culture or gender’
Belonging	Rootedness, a sense of place and belonging^28^Connectedness, including a feeling of belonging and identification with an area as an important aspect of personal identity^27^	‘It is really damaging for your mental health if you are really stressed and anxious about what's going on around where you live … ’
Social support	Perceived social support, includes social responsibility and solidarity^27^Trust, help, norms of reciprocity and cooperation^9,29^	‘We need that mutual trust to start anything within the community … ’‘To know that there are people that are looking out for you’
Shared values	Shared values to identify and achieve joint goals and objectives^27^There is a set of common attitudes and norms^29^	‘To see the community come together [..] and see them make decisions about where we live’
The (built)environment	Indirect aspects of neighbourhood social cohesion related to the physical and social environment Neighbourhoods shaped by physical environment (i.e. bicycle lanes) and urban planning decisions^11^ Presence of institutions of conflict management^9^	‘Community spaces where you can socialise with the community … it's really helpful when you're going through tough times … ’
Influence and participation	Political and socio-political participation^27^ The power of member and community to affect each other^30^	‘Looking out for your neighbours’ ‘Everyone's parents would look out for the kids even they weren't theirs’

The second workshop aimed to inform what future interventions to increase neighbourhood social cohesion may look like. We asked young people about what they felt could increase connection within their community (see Supplementary File 4 for the topic guide). A qualitative thematic analysis and a narrative literature review (separate to the rapid review) were conducted to identify any relevant themes and evidence related to potential interventions that may increase connection. The results of the workshops and literature review are presented in the ‘Future Interventions' section of the results. The workshops were held as consultation events, so no ethics approval was obtained. Verbal consent was sought at the start of the workshop.

## Results

After removing duplicates, 2261 title/abstracts were screened and 432 papers were included for full-text review. Forty-two peer-reviewed publications were identified, comprising two systematic reviews, 13 longitudinal studies and 27 cross-sectional studies (see Supplementary File 5 for the PRISMA-P flowchart). No intervention studies were identified (randomised controlled trials) that met our inclusion criteria. Most of the studies were conducted in North America (34 studies), followed by Asia, Australasia and Europe (non-UK) (two studies from each location) and UK (one study). Cities were the most often evaluated type of community/neighbourhood (22 studies). Table 2 provides an overview of the baseline characteristics of the included longitudinal studies (Supplementary File 6 shows baseline characteristics and results of cross-sectional and systematic reviews). We prioritised describing longitudinal results as this provides the most insight into any potential causal link between exposure to cohesion and mental health outcomes. All longitudinal studies were single-arm and non-comparative. The risk of bias across longitudinal studies was moderate to high, as studies often lacked detail on inclusion criteria and baseline characteristics (see Supplementary File 7 for quality assessment ratings of included studies). Still, most studies appeared to have sufficient sample sizes and were based on established longitudinal surveys. As none of the studies included randomisation, it is impossible to rule out that associations could be attributed to unobserved characteristics rather than neighbourhood social cohesion.^5^

**Table 2:** Baseline characteristics of included longitudinal studies

Reference	Source of data	Study location; type of community/ neighbourhood	Period of data collection or follow-up	Sample description: number of participants; age, mean (s.d., range); gender, %; ethnicity/race, %	Outcome measure
Abada et al, 2007^31^	The National Longitudinal Survey of Children and Youth (NLSCY)	North America; not reported	Wave 2: 1996–1997 Wave 4: 2000–2001	Wave 2: *n* = 1111; not reported (12–13 years); male 48.4%, female 51.6%; not reported, racial minorities in census tract 9.1% Wave 4: *n* = 1111; not reported (16–17 years); male 48.4%, female 51.6%; not reported, racial minorities in census tract 9.1%	Waves 2 and 4: child reported 12-item version of the CES-D
Basáñez et al, 2013^32^	Reteniendo y Entendiendo la Diversidad (RED)	North America; city	9th grade: 2005 11th grade: 2006	9th grade: *n* = 1045; not reported (14–15 years); male 45.7%, female 54.2%; Hispanic 100% 11th grade: *n* = 1045; not reported (16–17 years); male 45.7%, female 54.2%; Hispanic 100%	9th and 11th grade: child-reported 20-item version of the CES-D
Cerdá et al, 2011^33^	The Project on Human Development in Chicago Neighbourhoods Longitudinal Cohort Study (PHCDN)	North America; city	Wave 1: 1995–1997 Wave 2: 1997–2000 Wave 3: 2000–2002	Wave 1: *n* = 1517; 12.1 years (not reported, 54.1% of cohort, 12–15 years) and 15.2 years (not reported, 45.9% of cohort, 12–15 years); male 49%, female 51%; Black non-Hispanic 34%, White non-Hispanic 13.9%, Hispanic 40.4%, other 11.7% Wave 2: *n* = 1315; 14.2 years (not reported, 14–17 years) and 17.3 years (not reported, 14–17 years); not reported Wave 3: *n* = 1210; 16.8 years (not reported, 17–20 years) and 19.8 years (not reported, 17–20 years); not reported	Waves 2 and 3: child-reported MDD instrument adapted from the DISC 4
Donnelly et al, 2016^5^	The National Longitudinal Study of Adolescent Health (AddHealth)	North America; not reported	1999–2016	Overall: *n* = 2264; 15 years; male 52%, female 48%; non-Hispanic White 23%, non-Hispanic Black 50%, non-Hispanic other 4%, Hispanic (any race) 23%	Overall: child-reported five-item version of the CES-D; six-item version of the BSI
Estrada-Martínez et al, 2012^37^	The Flint Adolescent Study	North America; city	Wave 1: 1994 Wave 5: 1999 Wave 8: 2003	Wave 1: *n* = 604; 14.8 years (0.64); male 47%, female 53%; African American 100% Wave 5: *n* = 604; 20 years (not reported, 19–22 years); male 47%, female 53%; African American 100% Wave 8: *n* = 604; 23 years (not reported, 22–25 years); male 47%, female 53%; African American 100%	Waves 1, 5 and 8: child-reported six-item version of the BSI
Fredricks and Eccles, 2006^36^	The Maryland Adolescent Development in Context Study (MADICS)	North America; county	Wave 3: 8th grade; 1993 Wave 4: 11th grade; 1997 Wave 5: 1 year after high school; 1999	Overall: not reported (waves 3, 4 and 5: *n* = 1060, 1075 and 912, respectively); not reported (8th and 11th grades, 1 year after high school); male 49%, female 51%; African American 67%, European American 33%	Waves 3 and 4: child-reported 14-item CDI; parent-reported Child Behaviour Checklist Wave 5: child-reported six-item version of the CDI
Hurd et al, 2013^39^	The longitudinal study of factors that contribute to high school drop-out rates (title not reported)	North America; city	Waves 4–8: 1998–2003	Overall (waves 4–8): *n* = 570; 17.8 years (0.65) at wave 4; male 48%, female 52%; African American or Black 100%	Waves 4–8: child-reported six-item version of the BSI
Lee and Liechty, 2015^40^	The National Longitudinal Study of Adolescent Health (AddHealth)	North America; not reported	Wave 1: 1994–1995 Wave 2: approximately 1 year later	Latin American immigrant: Wave 1: *n* = 1114; 16.6 years (1.6, 12–20 years); male 49.7%, female 50.3%; Hispanic or Latin American 100% Wave 2: *n* = 1114; not reported; male 49.7%, female 50.3%; Hispanic or Latin American 100% Latin American non-immigrant: Wave 1: *n* = 1564; 16.2 years (1.8, 12–20 years); male 51.2%, female 48.8%; Hispanic or Latin American 100% Wave 2: *n* = 1564; not reported; male 51.2%, female 48.8%; Hispanic or Latin American 100%	Latin American immigrant and non-immigrant, waves 1 and 2: child-reported 19-item version of the CES-D
Solmi et al, 2017^41^	The Avon Longitudinal Study of Parents and Children (ALSPAC)	UK; province/county	from 1 April 1991 until child is 18 years old	Age 13 years: *n* = 6378; 13 years; male 49.1%, female 50.9%; White 96.2%, Black and minority ethnic 3.8% Age 18 years: *n* = 3231; 18 years old; male 36%, female 64%; White 96.3%, Black and minority ethnic 3.7%	Ages 13 and 18 years: child-reported 13-item version of the S-MFQ
Viau et al, 2015^42^	Longitudinal study on French-speaking youth recruited from schools in Canada	North America; province/county	Phase 1: 2001 (6th grade) Phase 2: 8th to 11th grade Phase 3: 2007 (1-year after high school)	Phase 1: *n* = 228; 12.38 years (0.42; 12 years); male 35%, female 65%; mostly White (percentage not reported) Phase 2: *n* = 228; 14–17 years; not reported; not reported Phase 3: *n* = 228; 18 years; not reported; not reported	Phase 1: child-reported French 26-item version of the CDI Phase 3: child-reported French 20-item version of the CES-D
Wu et al, 2010^45^	China Seven Cities Study (CSCS)	Asia; city	Since 2002 at 1-year intervals (3 years of follow-up)	Overall (waves 1–3): *n* = 5164; 15.45 years (1.69, 11–19 years); male 48.8%, female 51.2%; Chinese 100%	Wave 3 only: child reported Chinese version of the CES-D
Everett, 2014^46^	National Longitudinal Study of Adolescent Health (AddHealth)	USA adolescents	Wave 1: 1994 Wave 3: 2001	Overall (waves 1–3): *n* = 1328; 16.2 years (not reported); male 28%, female 72%; White 75%, Latin American 12%, Black 8%, Asian 3%, other 2%	Wave 3: CES-D
Burton et al, 2013^47^	Youth recruited from adolescent medicine clinics	North America; city	Wave 1: not specified Wave 2: approximately 6 months later	Overall (waves 1–3) for sexual minority youth: *n* = 55; 17.0 years (1.36); male 15%, female 85%; Black and minority ethnic 78%, White 22%	Wave 2: CES-D

CES-D, Center for Epidemiologic Studies Depression Scale; MDD, major depressive disorder; DISC 4, Depression Intensity Scale Circles; BSI, Brief Symptom Inventory; CDI, Children's Depression Inventory; S-MFQ, Short Moods and Feelings Questionnaire.

### Factors of neighbourhood social cohesion

Table 1 provides the theoretical conceptual framework of neighbourhood social cohesion. Seven neighbourhood social cohesion factors were identified from our thematic analysis: relationships, safety, belonging, social support, shared values, the (built) environment, and influence and social participation. An overview of definitions and illustrative quotes is further provided.

The study results in Table 3 show that there is evidence to support that factors of neighbourhood social cohesion are associated with mental health symptoms. Table 3 provides an overview of the neighbourhood social cohesion factor as identified in the theoretical conceptual framework, how it is measured, and the effect of exposure to this factor on depression and anxiety in young people. It appears that studies most consistently show that communities that have the presence of positive relationships and a lack of latent social conflict (i.e. safety) were most strongly associated with mental health symptoms. The below paragraphs describe which neighbourhood social cohesion factors were associated with symptoms of depression and anxiety.

**Table 3:** Neighbourhood social factors and their association with depression and anxiety in adolescents and young adults

Study	Evaluated neighbourhood social cohesion factor	Measurement of neighbourhood social cohesion	Results
Abada et al, 2007^31^	Relationships (i.e. whether neighbours know and help each other and are friendly) Safety (i.e. neighbourhoods that feel safe during daylight and after work) Belonging (i.e. having a positive role model) The (built) environment (i.e. ethnic composition, income, urbanicity)	Perceived neighbourhood social cohesion: the six-item questionnaire measuring the extent to which the respondents perceives the neighbourhood to be safe, during daylight and after work, whether neighbours know and help each other, are friendly and if there are adults that young people can look up to; reported by adolescents at cycle 4	Neighbourhood characteristics (ethnic composition, median income, living in major cities and other large urban areas) did not have an impact on the development of depressive symptoms in cycle 4 (*r*^2^ = 0.157, *P* > 0.05). The higher level of perceived neighbourhood cohesion is protective for the development of depressive symptoms at cycle 4 (*r* = −0.234, s.e. 0.091, *P* < 0.05)
Basáñez et al, 2013^32^	Safety (i.e. perceived discrimination) The (built) environment (i.e. neighbourhood composition)	Neighbourhood composition: information about the percentage of Hispanic living in the respondents’ neighbourhood was determined by linking zip codes to USA Census 2000 data	The effect of neighbourhood composition in 9th grade did not affect depression symptoms in 11th grade (β = 0.01, *P* = 0.73). The impact of interaction of neighbourhood composition and discrimination on depressive symptoms was at the borderline of significance (β = −0.07, *P* = 0.06)
Cerdá et al, 2011^33^	Safety (i.e. victimisation, including because of race, violence, harassment and exposure to violence)	Victimisation: participants were asked whether they had been attacked with a weapon, beaten up, chased, shot at, sexually assaulted or threatened with serious harm in the past year; reported at wave 2 Witnessing violence: questions adapted from ‘My Exposure to Violence’ (Selner-O'Hagan 1998^34^); reported by participants at wave 2	Odds of depression at wave 3 following victimisation and witnessing violence (past year) was 1.28 (95% CI 0.89–1.57, *P* = not reported) and 1.27 (95% CI 0.81–2, *P* = not reported), respectively
Donnelly et al, 2016^5^	Social control (i.e. whether parents would get involved in children misbehaving in a community) Relationships (i.e. neighbourhoods that people report as being close-knit and where generally people get along with each other) Safety (i.e. perceived safety) Shared values (i.e. sharing similar values in a community)	Exposure to neighbourhood collective efficacy: participant assessment using two adapted subscales derived from Morenoff et al, 2001^35^	Higher neighbourhood collective efficacy (combined) seems to have a protective effect on the development of depressive and anxiety symptoms in adolescents (age 15 years) (β = −0.114, s.e. 0.021, *P* < 0.01 for both comparisons) even after controlling sociodemographic variables and previous mental health history (β = −0.073, s.e. 0.023, *P* < 0.01; and β = −0.072, s.e. 0.023, *P* < 0.01 for depressive and anxiety symptoms, respectively)
Fredricks and Eccles, 2006^36^	Influence and participation (i.e. afterschool activities, including volunteering)	Extracurricular participation: assessed at 11th grade by participants; report on involvement in a range of activities such as school clubs (one question), organised sport involvement (two questions), volunteer service or civil rights activities (combined into prosocial activities; one question)	Participants taking part in sport activities showed significantly lower levels of depression as reported in 11th grade (F(8,733) = 10.03, partial η^2^ = 0.013, *P* < 0.01); however, the effect was not significant for adolescents attending school clubs and prosocial activities (F = 1.24, partial η^2^ = 0.002 and F = 2.07, partial η^2^ = 0.000, respectively; *P* > 0.05 for both). The effect of school clubs, sport activities or prosocial activities on the levels of depression was not significant at 1 year after high school (F = 0.44, partial η^2^= 0.001; F = 2.26, partial η^2^ = 0.005; F = 0.14, partial η^2^ = 0.0001, respectively; *P* > 0.05 for all comparisons). The breadth of participation in 11th grade or 1 year after high school was not associated with depression (β = −0.02 and −0.03, respectively; *P* > 0.05 for both)
Estrada-Martínez et al, 2012^37^	Safety (i.e. victimisation, including because of race, violence, harassment and exposure to violence and perceived discrimination) Social support (i.e. neighbours would help in an emergency)	Attitudes measured with five items and fear of violence measured with two items modified from Sampson and Wooldredge's (1987)^38^ measure of social cohesion and trust reported by participants at waves 5–8	The correlation between the neighbourhood stress and the risk of developing depressive symptoms over time was significant (β = 0.09, s.e. 0.02, *P* < 0.001)
Hurd et al, 2013^39^	The (built) environment (i.e. neighbourhood composition)	Racial composition: assessed at the block group level with 2000 USA Census information	The neighbourhood racial composition was not predictive of participants depressive symptoms at wave 4 (t(109) = −0.75, *P =* 0.455). Neighbourhood racial composition moderated the effect of higher levels of public regard (individuals’ perceptions of how other groups view their racial group) in predicting more symptoms of depression (t(109) = −2.44, *P* = 0.016)
Lee and Liechty, 2015^40^	Relationships (i.e. knowing most people in your neighbourhood) Influence (i.e. informal social control, whether you would tell if a neighbour's child got in trouble or *vice versa*)	Neighbourhood collective efficacy: five items reported by parents, pertaining to social cohesion (three items) and informal social control (two items)	The neighbourhood collective efficacy (immigrant: odds ratio 1.07, 95% CI 0.88–1.3, *P* > 0.05; and non-immigrant: odds ratio 0.94, 95% CI 0.75–1.17, *P* > 0.05) did not affect the odds of depression at wave 2
	Safety (i.e. whether you feel safe in the neighbourhood)	Participants’ reported perceived safety (one item) and perceived contentment (two items)	Perceived neighbourhood contentment (immigrant: odds ratio 1.06, 95% CI 0.75–1.5, *P* > 0.05; and non-immigrant: odds ratio 1.16, 95% CI 0.89–1.51, *P* > 0.05) or perceived neighbourhood safety (immigrant: odds ratio 1.33, 95% CI 0.58–3.09, *P* > 0.05; and non-immigrant: odds ratio 0.82, 95% CI 0.37–1.77, *P* > 0.05) did not affect the odds of depression at wave 2
	The (built) environment (i.e. neighbourhood composition and neighbourhood poverty)	Latin American immigrant density indicator was formed from three items in AddHealth's contextual variables, based on the 1990 USA Census set of 17 questions reported by mothers during pregnancy, at 8 months’ postpartum and approximately at child ages 2, 3, 4, 7 and 10 years	The effect of Latin American immigrant density on the odds of depression at wave 2 for immigrants and non-immigrants was 0.63 (95% CI 0.46–0.87, *P* < 0.01) and 0.82 (95% CI 0.66–1.02, *P* > 0.05). The effect of poverty on the odds of depression at wave 2 for immigrants and non-immigrants was 1.34 (95% CI 1.02–1.77, *P* < 0.01) and 1.27 (95% CI 1.03–0.56, *P* < 0.01)
Solmi et al, 2017^41^	Relationships (i.e. neighbourhood cohesion, whether people in your neighbourhood visit your home) Social support (i.e. neighbourhood trust, whether neighbours look after your children and *vice versa*)	Set of 17 questions reported by mothers during pregnancy, at 8 months’ postpartum and approximately at child ages 2, 3, 5, 7 and 10 years, evaluating neighbourhood social cohesion and trust, neighbourhood discord and neighbourhood stress/quality	The odds of depressive symptoms at 18 years in neighbourhood with medium and low neighbourhood cohesion was 1.15 (95% CI 0.92–1.43, *P* > 0.05) and 1.43 (95% CI 1.02–1.71, *P* < 0.05), respectively.
	Safety (i.e. neighbourhood discord; arguing with neighbours and neighbourhood stress; worrying about vandalism, burglaries, mugging or attacks)		The odds of depressive symptoms at 18 years in neighbourhood with medium and high neighbourhood discord was 1 (95% CI 0.82–1.24, *P* > 0.05) and 1.18 (95% CI 0.88–1.56, *P* > 0.05), respectively. The odds of depressive symptoms at 18 years in neighbourhood with medium and low neighbourhood stress was 1.24 (95% CI 1.02–1.5, *P* < 0.05) and 1.55 (95% CI 1.07–2.26, *P* < 0.05), respectively
Viau et al, 2015^42^	Influence and participation (i.e. intensity, duration and breadth of community activity)	Adolescents reported the number of different activity types (breadth of participation), the average intensity of participation in activities and the combined number of years that they participated in activities (the duration) from grades 8 to 11	The duration of participation in organised activities at ages 14–17 years, but not breadth or intensity, was significantly correlated with lower depressive symptoms at age 18 years (duration: β = −0.14, *P* < 0.05; breadth: β = −0.07, *P >* 0.05; intensity: β = −0.04, *P >* 0.05)
	Social support (i.e. support from activity leader) Relationships (i.e. social integration; ‘I feel appreciated by the other kids’, ‘I am quite alone and don't talk to anyone’)	Support from activity leader: adapted four-item scale from Mahoney et al (2002)^43^, reported by adolescents Social integration: adapted five-item scale from Denault and Poulin (2008)^44^, reported by adolescents	The social integration into peer group was an indirect mediator between the duration of participation and depressive symptoms (β = −0.02, s.e. 0.01, *P* < 0.05), whereas support from activity leader did not mediate this correlation (β = −0.01, s.e. 0.01, *P >* 0.05). The effect was significant for boys (β = −0.1, s.e. 0.03 *P =* 0.002), but not for girls (β = −0.01, s.e. 0.01 *P =* 0.261)
Wu et al, 2010^45^	Safety (i.e. do you feel safe in your neighbourhood?)	Five-item questionnaire regarding community social capital assessed by participants and their parents	Significant and negative correlation was reported for depressive symptoms and neighbourhood safety (*r* = −0.128, *P* < 0.05).
	Social support (i.e. do neighbours care about how things are going in your life) Relationships (i.e. how many friends live in your neighbourhood)		Significant and negative correlation was reported for depressive symptoms and degree of neighbours caring about one's life (*r* = −0.157, *P* < 0.05). Higher levels of social capital were associated with lower level of depressive symptoms (β = −0.097, *P* < 0.001)
Everett, 2014^46^	The (built) environment (i.e. urbanicity)	Urbanicity (percentage) was assessed with USA Census data	Urbanicity, but not change in neighbourhood urbanicity over time, was associated with depressive symptoms. With lower urbanicity being associated with higher depressive symptoms (β = 0.09, s.e. 0.04, *P* < 0.05) for sexual minority young adults
Burton et al, 2013^47^	Safety (i.e. victimisation, including because of race, violence, harassment and exposure to violence)	Four items assessing victimisation (i.e. frequency of bullying, hit/beaten up, treated unfairly or called bad names because someone thought the participant was gay/lesbian)	Sexual-minority-specific victimisation at wave 2 significantly mediated the effect of sexual minority status on depressive symptoms at wave 2 (β = 0.045, 95% CI 0.0063–0.15)

#### Relationships, social support, influence and belonging

Two studies found that increased levels of positive social relationships in the community were associated with reduced levels of depression at follow-up. Solmi et al^41^ found that low levels of neighbourhood interaction (positive relationships) and social support (including trust) reported by parents of children at 13 years of age were associated with an increased risk of depressive symptoms at 18 years of age. Children exposed to high neighbourhood collective efficacy (cooperative behaviour, shared values, close relationships and social control) experienced lower depression and anxiety symptoms at 15 years of age compared with adolescents growing up in a low collective efficacy neighbourhood.^5^

#### Relationships and safety

Five studies found evidence for the effects of positive relationships, social support and a lack of latent social conflict (i.e. safety) on mental health. Wu et al^45^ studied neighbourhood social cohesion, family capital and financial capital among 5164 adolescents. Factors of neighbourhood safety and social support (i.e. whether neighbours care about other neighbours) were associated with lower levels of depressive symptoms at 3-year follow-up. Similarly, Estrada-Martínez et al^37^ found that increased fear of violence and lower levels of trust and social support were associated with higher depressive symptoms over time. The presence of a positive role model, alongside safety, social support and positive relationships, was also associated with reduced depressive symptoms in adolescents in a longitudinal cohort study in Canada.^31^

#### Influence and participation

Social and political participation at the community level includes taking part in locally organised activities such as community groups and sports, as well as activism to affect local and national policy issues.^48^ Of all extracurricular or community participation for adolescents, engaging in group sports seems to be the only activity that has thus far been associated with reduced depressive symptoms. Viau et al^42^ studied whether participation in cultural, sporting (individual and team) and civic activities, as well as the extent thereof (support from activity leaders, social integration), between ages 14 and 17 years was associated with reduced depressive symptoms at age 18 years in adolescents. Only in the team sports activity group was an association found: the more boys felt integrated into the community peer group (social support), the longer they engaged in activities and the fewer depressive symptoms they reported. Team sports were also available for girls, but there was a lower uptake. Potentially, team sports can be made more attractive, and alternative forms of active participation might be more beneficial for girls.^42^

#### Neighbourhood safety

The absence of latent social conflict comprises whether communities are safe and there is little violence.^9^ Only one study evaluated this construct separately, and found that lower levels of perceived safety at age 13 years were associated with higher depressive symptoms at age 18 years.^41^

#### Violence

Young people may be exposed directly to violence in the community, as well as indirectly through parental stress from perceived social disorder.^49^ Wu et al^45^ noted an indirect effect of violence on mental health symptoms, namely living in safety relieves parents from stressors, allowing them to spend more time with their children and focus on the child.^45^

Direct exposure to violence may also be associated with the occurrence of depression, although the evidence is mixed for adolescents and young adults. On the one hand, Cerdá et al^33^ found that among adolescents, violent victimisation and witnessing violence were not associated with an increase in depressive symptoms. On the other hand, self-reported victimisation based on sexual orientation or sexual minority and harassment was associated with depressive symptoms in LGBTQ adolescents and young adults.^47,50^ Similarly, perceived racial discrimination in the neighbourhood has been associated with increased symptoms of depression in two longitudinal studies among adolescents^32^ and young adults.^37^

#### The (built) environment

Included studies primarily evaluated neighbourhood racial composition as an environmental factor. There was no evidence to suggest that neighbourhood composition was associated with depressive symptoms in two longitudinal surveys.^32,39^ One longitudinal study did suggest that neighbourhood composition may only be affecting immigrants in contrast to non-immigrants.^40^ Lee and Liechty found that, among 2678 Hispanic youth, Latin American immigrant density was associated with lower odds of depression for immigrants, but this association was not found for non-immigrant Latin American adolescents.^40^ In contrast, a longitudinal study among Canadian adolescents found that visible minority youth living in areas with a high concentration of minorities had higher symptoms of depression compared with visible minority youth living in largely White neighbourhoods.^31^ As the evidence is mixed in this area, further research is needed to unravel drivers for increased risk of depressive symptoms.

Living in large cities did not seem to affect the development of depressive symptoms in one longitudinal study among Canadian adolescents.^31^ However, urbanisation may even be protective for some: a longitudinal study in the USA found that that decreases in urbanisation were associated with higher depressive symptoms for sexual minority youth.^46^

To summarise, neighbourhood factors, including positive relationships and a lack of social conflict and violence, are associated with decreased depressive symptoms, and in one case, symptoms of anxiety. The environmental factors that were studied (neighbourhood composition and urbanicity) show mixed effects. It appears urbanisation could be protective for sexual minority youth, although further research is needed.

### Future interventions

No intervention studies that met our inclusion criteria were identified for the rapid review. To inform future interventions and what they could look like, we provide an overview of future interventions to increase neighbourhood social cohesion. These interventions are suggested based on lived experience workshops and a narrative literature review. An overview of the results of the lived experience workshops can be found in Supplemental File 8, and we provide quotes from the workshops.

#### Volunteering

One potential avenue to improve neighbourhood social cohesion is increasing community activity in the form of community groups or volunteering. For instance, Ohmer^51^ found that volunteering in community groups organised through small non-profit organisations (i.e. neighbourhood beautification, crime prevention and leadership development) was associated with an increased sense of community, self-efficacy and community efficacy for people taking part in the groups.^51^ Large-scale national volunteering programmes (i.e. National Citizen Service (NCS), England) for adolescents and young adults (aged 15–17 years) have also found that community involvement and social cohesion increased compared with control groups (participants who expressed interest in taking part in a NCS intervention but did not participate). A quasi-experimental study to evaluate the effects of the NCS (6-week intervention comprising community awareness and planning and delivery of a social action project in the local community) found that local community involvement increased, as did feelings of cohesion versus control. Although the effects on mental health were not measured with a validated measure of depression or anxiety to ascertain its potential preventive effects, a self-report item of ‘% who did not feel anxious at all yesterday’ and increased personal resilience were observed among young people taking part in the NCS (*n* = 1608) compared with the matched control group (*n* = 2041) at 3- to 5-month follow-up.^52^ In the workshops, adolescents and young adults also highlighted how volunteering can help provide a sense of cohesion:

‘Joining the community and getting involved, if we got people to volunteer … get them to fundraise … volunteering in the community is a corner stone to getting to know people and getting sociable.’

#### Arts, creativity and culture

Arts and culture may increase positive relationships and participation in neighbourhood activities. Generally, the more arts and cultural activities available in a community, the more participation in community cultural activities can be observed.^53^ Using arts as a community intervention to improve adolescent and young adult mental health may be promising, although most evidence is qualitative.^54^ For example, Fanian et al^55^ studied creative arts projects where five workshops with five to six youth were held to explore community issues and find solutions through the arts (music and film). Initial qualitative reports suggest that the groups helped build positive relationships and empowerment to influence change. From our workshops, the adolescents and young adults felt that having community spaces that provide activity groups would improve connection and a sense of belonging. Finding creative ways to bring these arts and culture groups to the community was important:

‘I think it was a charity that had a minibus that had equipment for people to write music … they had a studio … even if we can't get the funding to create a whole new building, how we can try and find ways to bring different activities for people to get involved in within the community.’

#### Sport groups

Participating in organised group sports activities can be a helpful pathway to form positive networks with peers and adults.^56^ Most studies evaluating sports have been conducted in a school rather than neighbourhood context. Langbein and Bess (2002) found that organised sports can help to reduce school disturbances, highlighting that is some preliminary evidence to suggest that organised sports may be effective in this setting to decrease social conflict, by creating the opportunity for repeated social interaction to help reinforce social norms and reduce the development of conflict.^56^

‘A big park … there's a lot of cohesion with different people doing different sports, being able to share it.’

#### Online spaces

Online platforms or networks can be another avenue to foster (neighbourhood) social cohesion, especially with the COVID-19 pandemic limiting opportunities to meet face to face. Some preliminary qualitative evidence suggests that using social media sites can be helpful to facilitate community interaction and increase participation in (offline) sports activities.^57^ To build successful engagement, it has been suggested that such platforms are best created bottom-up (by the community) and rely on community members to maintain and promote the platform to enhance engagement from the community.^58^ In the workshops, adolescents and young people also suggested that online community groups on Facebook and WhatsApp could increase social support in the neighbourhood through offering social support and increased social interaction:

‘A really big Facebook group page, everyday everyone's putting on “who's lost this” … being really honest with each other.’

#### Green space

Increasing green space may provide more opportunities to build relationships with neighbours, in turn increasing neighbourhood social cohesion. For adolescents and young adults (aged 13–19 years), a rapid review observed that adding more natural elements and opportunities for play in outdoor spaces was associated with increased social connectedness.^59^ A systematic review looking at the fear of crime in urban green spaces found that vandalised or run-down parks are associated with higher levels of crime, and that this fear of crime in poorly maintained green spaces disproportionately affects women, girls and ethnic minorities in terms of their mobility and feelings of safety.^60^ In the workshops, adolescents and young people emphasised the opportunity of parks for exposure to, and interaction with, different communities and generations:

‘A big park … centre of the community, it's got a children's play area and a big field with football goalposts and a nice hill which is nice to sit on … the fact that these green spaces can be used by multiple different people … that park has been the centre of the community since it was a field in the 60s.’

#### Neighbourhood regeneration programmes

Neighbourhood regeneration programmes are frequently seen as an opportunity to improve the built environment, which can result in improved social benefits.^61^ Overall, evidence suggests that changes in the built environment via neighbourhood regeneration programmes can be beneficial for mental health in adults. For example, in Communities First, a neighbourhood intervention across deprived neighbourhoods in Wales, White et al^61^ observed a significant reduction in mental health symptoms in the regeneration intervention group (composed mostly of building new community facilities) compared with the propensity-score-matched control group. Further research on this programme suggests that the effect on mental health occurred through increased neighbourhood quality and reduced disorder,^62^ which might be valuable target points for future interventions. Alongside parks and spaces, structural elements such as transportation should also be considered for neighbourhood social cohesion. Hart and Parkhurst^63^ observed that where streets had higher volumes of motor traffic, residents reported fewer friends and acquaintances on the street. In the workshops, it was emphasised that adolescents and young people would be keen to have a say in future developments; for example, by joining council meetings:

‘To focus on the people and get people involved so they themselves feel they are valued … more community representatives that can get involved in council meetings … more responsibility and [a] more connected community.’

#### Psychosocial interventions

Community groups may increase neighbourhood social cohesion by creating repeated opportunities to connect and share experiences. For example, a quasi-experimental study in Rwanda studied the effects of a group intervention aiming to increase social capital (measuring cognitive social capital), support (receiving financial, instrumental or emotional support) and civic participation (joining in addressing local issues and meetings, engaging with selecting local leadership) in an adult population. It found that, among 200 adults, the intervention had a positive effect on both mental health and civic participation compared with a matched control group, but not on cognitive social capital and support over a 15-month follow-up.^64^ Moreover, community-based groups aimed at empowering and teaching life skills to young women in India were associated with reduced depressive and anxiety symptoms and a shift in community attitudes toward violence against women and girls.^65^ Considering gender dynamics when developing community group interventions for social capital is important, as participation in groups can reinforce gender inequality with negative consequences for the mental health of women.^66^ In the workshops, it was highlighted that a community centre to host community groups would be beneficial, as it offered different integrations between people who would not usually connect. Particularly, offering an area like a café might offer opportunities for connection between community groups:

‘What I liked about it is that you get all these different people, you had a group for people with mental health difficulties, a group for people with disabilities … you did see some integration of people that don't usually meet each other.’

#### Adverse effects

Neighbourhood social cohesion may also have adverse effects. For example, people who move into a community may be excluded if the existing social networks are highly cohesive.^67^ Second, high levels of social control and observation may also limit young people's exploration and mobility^67^ and contribute toward anxiety. Indeed, adolescents in London indicated that they prefer some level of social control (i.e. willingness of neighbours to intervene with challenges) and proximity of caregivers (indirect observation), but not direct control and observation from caregivers or parents.^28^ This means that when designing interventions, the current level of social cohesiveness of a neighbourhood should be considered, especially levels of social control and observation.

## Discussion

### Summary of evidence

We set out to study which elements of neighbourhood social cohesion may be preventive for depression and anxiety in adolescents and young adults, whether any interventions that increase cohesion can be effective and what the views of young people were on increasing neighbourhood social connection and what future interventions may look like. In consultation with young people, we developed a conceptual theoretical framework on the factors that comprise neighbourhood social cohesion. We identified seven factors, namely relationships, safety, belonging, social support, shared values, the (built) environment and influence.

We then explored whether neighbourhood social cohesion factors were associated with depression and anxiety in adolescence and young adulthood. Positive relationships, social support, the ability to enforce social norms, feeling safe, little nuisance and antisocial behaviour, and trusting others in the neighbourhood were associated with fewer symptoms of depression and anxiety in adolescents and young adults.^31,37,39,41,45^ Neighbourhood violence, which may include victimisation and harassment, was also associated with more depressive and anxiety symptoms in a number of longitudinal studies and systematic reviews included in this review.^45,50,68^ Thus, the ability to change these factors may all be potential target points for interventions that reduce the risk of developing depression and anxiety.

Next, we studied whether there is evidence for interventions aiming to increase neighbourhood social cohesion and what future interventions could look like. We found no experimental or quasi-experimental studies evaluating the preventive effect of neighbourhood social cohesion interventions on depression and anxiety in adolescents and young adults. As such, there is inconclusive evidence of an effect of interventions to prevent depression and anxiety. However, we describe several interventions that target neighbourhood social cohesion factors that were informed by young people, which can be used for future research. These include creating safe and inclusive community centres, accessible outdoor spaces, community groups and activities that foster engagement, and increasing the connection between online and offline activities in order to enhance further cohesion.

### Strengths

This is the first rapid review available on neighbourhood social cohesion for preventing depression and anxiety in adolescents and young people. The findings offer a building block for future research. We developed a theoretical framework. We identified the factors of neighbourhood social cohesion for which there is most evidence for a potential preventative effect. Then, we identified future intervention strategies that target the factors of neighbourhood social cohesion, which can be utilised for future research.

### Limitations

This review also has several limitations. First, only one researcher conducted the screening and extractions, with a second researcher cross-checking 20% of their work database for peer-reviewed papers (PubMed), which may have limited the results we identified. However, we supplemented this by searching reference lists of included papers and systematic reviews. We also conducted a thorough search of grey literature databases and an online search of interventions. We were furthermore unable to pool all of the data to conduct a meta-analysis, because of the heterogeneity of studies and reporting of study results.

### Future research

#### Improving opportunity for causal inference

Several reflections regarding the overall state of the literature can also be made. A key observation of research on neighbourhood social cohesion is the lack of intervention studies. As also evidenced by this review, most studies studying the effect of neighbourhood factors on health use cross-sectional designs and are at high risk of bias.^11^ This limits causal inference in studies of neighbourhoods and mental health.^11^ Future research should consider moving away from correlational studies to methods allowing for more causal inference, including longitudinal, change-on-change analytical, quasi-experimental and experimental study designs.

#### Improving measurement and analysis

The measurement tools used in the review studies were diverse: five of the longitudinal studies developed questionnaires^31,36,40,41,45^ and four used modified scales from previous studies.^5,33,37,42^ Most studies combined factors into one questionnaire. By combining multiple constructs into one tool, it is difficult to disentangle exact factors (i.e. safety, social support) of neighbourhood social cohesion and the relative effects it might have on mental health. We propose further work on individual measurement and analysis of the neighbourhood social cohesion factors identified in this review, rather than using composite measures of cohesion. This includes a separate search of elements of cohesion as identified by our theoretical framework, rather than searching only on neighbourhood social cohesion. This will allow researchers to understand more about the relative contribution of separate factors of neighbourhood cohesion and their potential interactions over time.

Neighbourhood cohesion is a construct that is best conceptualised at the community level. However, in practice, it is often measured and modelled at an individual level. Measuring a community construct at an individual level may introduce bias, especially in studies of depression. Spurious relations may arise if the informant with depression is the same as the informant on positive relationships or the perception of safety. Individual characteristics, such as negative mood or a tendency to give extreme answers, may bias associations (shared method variance bias). Improving our ability to measure neighbourhood social cohesion at a community level and making data-sets available that have community-level indicators for social cohesion are both needed in future research.

System dynamics modelling can be a future avenue to distinguish both individual- and community-level contributions of neighbourhood social cohesion to mental health. In system dynamics modelling, individual, social and societal factors (and their interactions over time) can be mapped and investigated. Building a complex system model of neighbourhood social cohesion offers an opportunity to test and identify potential targets and the magnitude of the effect of interventions on a mental health system as a whole (for instance, on school drop-out rates and access to mental healthcare).

#### Improving generalisability

In our review, most studies in were conducted in North America. Notably, we did not identify a single study in a low- or middle-income country. It would be beneficial to see replications of the neighbourhood social cohesion factors in other areas. Anxiety outcome measures were also not included as frequently compared with depression outcome measurement tools: they were only reported in two out of 13 studies. We recommend further effort into studying these underresearched areas and groups, decreasing the risk of bias by improved reporting of baseline characteristics.

### Implications for clinical practice and policy

For clinical practice, these results suggest that clinicians should not only focus on the individual and family, but also consider interventions that enhance connections within the neighbourhood and actively seek existing activities in the neighbourhood for signposting. In policy development, it is important that future interventions involve co-production,^66^ and consideration is given to ensure inclusivity and accessibility, with differential effects on gender, disability and ethnicity considered.

To conclude, broadening current practice and research to include factors of neighbourhood social cohesion as identified in this review, has the potential to improve future prevention and research in depression and anxiety prevention in young people. Several approaches should be further explored and implemented, such as creating safe and attractive community centres, accessible and safe outdoor spaces, organising community activity groups and supporting engagement with online platforms to facilitate connections offline. In addition, clinicians must consider the community context alongside the individual and family context in their practice. Future interventions may benefit from the wide range of options available for increasing neighbourhood social cohesion, tailored to the neighbourhood and developed in collaboration with young people.

## Data Availability

The data that support the findings of this study are available from the corresponding author, J.J.F.B., upon request.

## Supplementary material

For supplementary material accompanying this paper visit http://doi.org/10.1192/bjo.2022.57. click here to view supplementary material
