# Supplementary material for: The effects of neighbourhood social cohesion on preventing depression and anxiety among adolescents and young adults: rapid review
Source: BJPsych Open. 2022 Jun 1;8(4):e97. doi: 10.1192/bjo.2022.57 (PMC9230698; doi:10.1192/bjo.2022.57)
Supplement: Supplementary file 1 [file S2056472422000576sup001.docx]

**Contents**

1. **Search strings**
2. **Protocol**
3. **PICOS table**
4. **Lived Experience Workshop outline**
5. **PRISMA-P Flowchart**
6. **Baseline characteristics and study results of cross-sectional studies and systematic reviews**
7. **Quality assessment**
8. **Workshop results**
9. **References**
10. **Search strings**

**Peer-review literature and grey literature**

The following databases and resources were searched:

- PubMed (https://pubmed.ncbi.nlm.nih.gov/)
- KSR Evidence (https://ksrevidence.com/)
- Campbell Collaboration (https://campbellcollaboration.org/better-evidence)
- Social Care Online (www.scie-socialcareonline.org.uk/)
- OAIster (http://oaister.worldcat.org/)
- OpenGrey (http://www.opengrey.eu/)
- Jisc Library Hub Discover (https://discover.libraryhub.jisc.ac.uk/)
- DH-DATA: Health Administration, Medical Toxicology & Environmental Health (Dialog)
- Google (https://www.google.co.uk/)

The following mental health and social care organisation websites were searched:

- Young Minds (https://youngminds.org.uk/)
- Centre for Mental Health (https://www.centreformentalhealth.org.uk/)
- Institute of Mental Health (https://www.institutemh.org.uk/)
- Joseph Rowntree Foundation (JRF)
- WHO Mental Health (https://www.who.int/mental_health/en/)
- US National Institute of Mental Health (NIMH) ([www.nimh.nih.gov](http://www.nimh.nih.gov))
- Mental Health Foundation (https://www.mentalhealth.org.uk/)

1. **Peer review database and grey literature resources searches**

**PubMed (https://pubmed.ncbi.nlm.nih.gov/): up to 24 June 2020**

**Searched: 24.6.20**

((((neighbourhood social cohesion) OR (social capital) OR (neighbourhood cohesion) OR (social cohesion) OR (community cohesion) OR (neighbourhood support) OR (neighbourhood networks)) AND ((prevention) OR (effects) OR (intervention) OR (public health)) AND ((youth) OR (young people) OR (children) OR (adolesc*) OR (students)) ) AND ((anxiety) OR (depression)))) Filters: Child: birth-18 years, Adolescent: 13-18 years, Young Adult: 19-24 years

**Records retrieved 1,597**

**KSR Evidence (Internet): database last updated Wed Jul 08 2020**

**www.ksrevidence.com**

**Searched: 9.7.20**

# Query Results

1 neighbourhood* or neighbourhood* in All text 66 results

2 "community cohesion" or "community connection" or "community connections" or "community connected" or "community network" or "community networks" or "community participation" or "community engagement" or "community engage" or "community engaged" in All text 132 results

3 "community belong" or "community belonging" or "community value" or "community trust" or "urban cohesion" or "residence characteristics" in All text 81 results

4 "social cohesion" or "social support" or "social capital" or "social network" or "social networks" or "social environment" or "socially integrate" or "social integration" in All text 1589 results

5 #1 or #2 or #3 or #4 in All text 1817 results

6 "mental health" or "mental illness" or "mental disorder" or "mental disorders" or "mental distress" or "mentally ill" or "mental ill-health" in All text 6502 results

7 anxiety or anxieties or depression or depressive or resilience in All text 10651 results

8 #7 or #6 in All text 14649 results

9 child or children or "young people" or "young adult" or "young adults" or youth or "early adult" or "early adults" or "emergent adult" or "emergent adults" or adolescen* or teenage* in All text 18203 results

**10 #5 and #8 and #9 in All text 165 results**

Database last updated Wed Jul 08 2020

**Campbell Collaboration (Internet): up to 9.7.20**

**https://campbellcollaboration.org/better-evidence**

**Searched 9 July 2020**

| **Search** | **Records found** |
| --- | --- |
| intext:neighbourhood OR intext:neighborhood | 2 |
| "social cohesion" | 0 |
| "community cohesion" | 1 |
| "social capital" | 2 |
| "social integration" OR "sociallly integrate" | 0 |
| "community participation" | 3 |
| "community involvement" | 0 |
| community engagement | 1 |
| **Total** | **9** |

**Social Care Online (Internet): up to 9.7.20**

**www.scie-socialcareonline.org.uk/**

**Searched 9 July 2020**

**Advanced Search; All Fields**

| **Search** | **Results** |
| --- | --- |
| neighbourhood AND "mental health" AND young | 37 |
| neighbourhood AND "mental health" AND youth | 27 |
| neighbourhood AND "mental health" AND children | 53 |
| neighbourhood AND "mental health" AND child | 44 |
| neighbourhood AND "mental health" AND adolescence | 28 |
| neighbourhood AND "mental health" AND teenage | 5 |
| neighbourhood AND depression AND young | 10 |
| neighbourhood AND depression AND youth | 6 |
| neighbourhood AND depression AND children | 19 |
| neighbourhood AND depression AND child | 13 |
| neighbourhood AND depression AND adolescence | 7 |
| neighbourhood AND depression AND teenage | 3 |
| neighbourhood AND anxiety AND young | 9 |
| neighbourhood AND anxiety AND youth | 3 |
| neighbourhood AND anxiety AND children | 8 |
| neighbourhood AND anxiety AND child | 7 |
| neighbourhood AND anxiety AND adolescence | 5 |
| neighbourhood AND anxiety AND teenage | 1 |
| "social cohesion" AND "mental health" | 11 |
| "social cohesion" AND depression | 5 |
| "social cohesion" AND anxiety | 0 |
| "community cohesion" AND "mental health" | 11 |
| "community cohesion" AND depression | 2 |
| "community cohesion" AND depression | 2 |
| "social capital" AND "mental health" AND young | 14 |
| "social capital" AND "mental health" AND youth | 7 |
| "social capital" AND "mental health" AND children | 18 |
| "social capital" AND "mental health" AND child | 6 |
| "social capital" AND "mental health" AND teenage | 1 |
| "social capital" AND depression AND young | 4 |
| "social capital" AND depression AND youth | 2 |
| "social capital" AND depression AND children | 4 |
| "social capital" AND depression AND child | 3 |
| "social capital" AND depression AND teenage | 0 |
| "social capital" AND anxiety | 6 |
| "social integration" AND "mental health" AND young | 6 |
| "social integration" AND "mental health" AND youth | 3 |
| "social integration" AND "mental health" AND children | 6 |
| "social integration" AND "mental health" AND child | 1 |
| "social integration" AND "mental health" AND teenage | 0 |
| "social integration" AND depression | 12 |
| "social integration" AND anxiety | 4 |
| "community participation" AND "mental health" AND young | 4 |
| "community participation" AND "mental health" AND youth | 1 |
| "community participation" AND "mental health" AND children | 4 |
| "community participation" AND "mental health" AND child | 3 |
| "community participation" AND "mental health" AND teenage | 0 |
| "community participation" AND depression | 11 |
| "community participation" AND anxiety | 5 |
| "community involvement" AND "mental health" | 15 |
| "community involvement" AND depression | 1 |
| "community involvement" AND anxiety | 0 |
| "community engagement" AND "mental health" AND young | 8 |
| "community engagement" AND "mental health" AND youth | 4 |
| "community engagement" AND "mental health" AND children | 6 |
| "community engagement" AND "mental health" AND child | 4 |
| "community engagement" AND "mental health" AND teenage | 0 |
| "community engagement" AND depression | 7 |
| "community engagement" AND anxiety | 1 |
| **Total** | **483** |
| **Total after removal of duplicates** | **230** |

**OAIster (Internet): up to 9.7.20**

**http://oaister.worldcat.org/**

**Searched 9 July 2020**

kw:("neighbourhood cohesion" OR "neighborhood cohesion" OR "social cohesion" OR "community cohesion" OR "community participation" OR "community involvement" OR "community engagement" OR "social capital" OR "social network*") kw:("mental health" OR "mental disorder*" OR "mental illness*" OR depression OR depressive OR anxiety OR anxieties OR resilience) kw:("young people" OR "young adult*" OR youth OR child OR children OR adolescen* OR teenage* OR "emergent adult*" or "early adult*")

7 records retrieved

**OpenGrey (Internet): up to 9.7.20**

**http://www.opengrey.eu/**

**Searched 9 July 20202**

| **Search** | **Results** |
| --- | --- |
| neighb* AND mental AND young | 2 |
| neighb* AND depress* | 14 |
| neighb* AND anxiety* | 6 |
| "social cohesion" AND mental | 1 |
| "social cohesion" AND depress* | 2 |
| "social cohesion" AND anxiety | 0 |
| "community cohesion" AND mental | 1 |
| "community cohesion" AND depress* | 0 |
| "community cohesion" AND anxiety | 0 |
| "social capital" AND mental | 11 |
| "social capital" AND depress* | 2 |
| "social capital" AND anxiety | 0 |
| **Total** | **39** |
| **Total after removal of duplicates and irrelevant records** | **20** |

**Jisc Library Hub Discover (Internet): up to 9.7.20**

[**https://discover.libraryhub.jisc.ac.uk/**](https://discover.libraryhub.jisc.ac.uk/)

**Searched 9 July 2020**

("neighbourhood cohesion" | "neighborhood cohesion" | "social cohesion" | "community cohesion")(mental | depression | anxiety) (young | youth | adolesc* | child*)

**18 records retrieved**

**DH-DATA: Health Administration, Medical Toxicology & Environmental Health (Dailog): 1983-2020 (current)**

**Searched 9.7.20**

S1 (neighbourhood* or neighbourhood*) 573

S2 ((community NEAR/3 (cohesion* or connection* or connected or network* or participation or engage* or belong* or value* or trust))) 787

S3 (social* NEAR/3 (cohesion or support or capital or network* or integrat*)) 2056

S4 S1 OR S2 OR S3 3249

S5 (mental health or mental illness or mental disorder* or mental distress or mentally ill or mental ill-health) 13376

S6 anxiety or anxieties or depression or depressive or resilience 4144

S7 S5 OR S6 15684

S8 S4 AND S7 737

S9 child or children or "young people" or "young adult" or "young adults" or youth or "early adult" or "early adults" or "emergent adult" or "emergent adults" or adolescen* or teenage* 18662

**S10 S8 AND S99 162**

**Google (Internet): up to 8.7.20**

**https://www.google.co.uk/**

**Searched _ July 2020**

neighbourhood cohesion young people mental health review report

social cohesion young people review report

community cohesion young people review report

community cohesion young people anxiety depression

community cohesion young people resilience

neighbourhood social community cohesion young people mental health anxiety depression resilience review report

neighbourhood social community cohesion young people anxiety depression resilience review report

neighbourhood social community cohesion young people adolescen* anxiety depression resilience review report

neighbourhood social community cohesion "young people" anxiety depression resilience review report

"social cohesion" neighbourhood community connection "young people" anxiety depression resilience review report

"social cohesion" neighbourhood community connection "young people" anxiety depression mental health report

"social cohesion" neighbourhood community cohesion connection "young people" anxiety depression resilience report

((neighbourhood OR community) AND ("social cohesion" OR cohesion)) AND ("young people" or adolescent or adolescence) AND (anxiety or depression or resilience) and report

"social cohesion" neighbourhood community cohesion connection "young people" anxiety depression resilience report

social community neighbourhood connection engagement young mental health experience

|  | **peer** | **reports** | **websites** | **other** |
| --- | --- | --- | --- | --- |
| **first 10** | **13** | **79** | **5** | **3** |

**2) Mental health and social care organisation websites searched**

Organisation websites were browsed (publications and/or research) and searched using a variety of combinations of the following terms: neighbourhood cohesion, social cohesion, community cohesion, social capital, social integration, community participation, community involvement, community engagementy, mental health, mental disorder(s), depression, anxiety, young people/adults, children, adolescence, teenage

Searches were conducted on 9-10 July 2020

**Search results**

**Table. Databases/resources searched**

| **Database/**  **Resource** | **Host** | **Date range** | **Results** | **Date Searched** |
| --- | --- | --- | --- | --- |
| PubMed | NLM | up to 24 June 2020 | 1597 | 24.6.20 |
| KSR Evidence | https://ksrevidence.com/ | database last updated Wed Jul 08 2020 | 165 | 9.7.20 |
| Campbell Collaboration | https://campbellcollaboration.org/better-evidence | up to 9 Jul 2020 | 9 | 9.7.20 |
| Social Care Online | www.scie-socialcareonline.org.uk/ | up to 9 Jul 2020 | 230 | 9.7.20 |
| OAIster | http://oaister.worldcat.org/ | up to 9 Jul 2020 | 6 | 9.7.20 |
| OpenGrey | http://www.opengrey.eu/ | up to 9 Jul 2020 | 15 | 9.7.20 |
| Jisc Library Hub Discover | https://discover.libraryhub.jisc.ac.uk/ | up to 9 Jul 2020 | 18 | 9.7.20 |
| DH-DATA: Health Administration, Medical Toxicology & Environmental Health | Dialog | 1993-2020 (current) | 162 | 9.7.20 |
| Google | https://www.google.co.uk/ |  | 84 | 8.7.20 |
| Mental Health Foundation | https://www.mentalhealth.org.uk/ |  | 3 | 9.7.20 |
| Young Minds | https://youngminds.org.uk/ |  | 0 | 9.7.20 |
| Centre for Mental Health | https://www.centreformentalhealth.org.uk/ |  | 2 | 10.7.20 |
| Institute of Mental Health | https://www.institutemh.org.uk/ |  | 0 | 10.7.20 |
| Joseph Rowntree Foundation (JRF) |  |  | 10 | 10.7.20 |
| WHO Mental Health | https://www.who.int/mental_health/en |  | 1 | 10.7.20 |
| US National Institute of Mental Health (NIMH) | www.nimh.nih.gov |  | 0 | 10.7.20 |
| **Total records retrieved** | | | **2302** | |
| **Duplicate records removed** | | | **1681 (PubMed/Google)**  **+**  **163 (KSR Evidence**  **+**  **304 (grey Lit)** | |
| **Total records screened** | | | **2148** | |

1. **Study protocol**

**Systematic Scoping Review – Wellcome Active Ingredient: increased neighbourhood cohesion**

| **Title** |
| --- |
| Can we prevent depression and anxiety in adolescents and young adults by increasing neighbourhood social connectedness? |
| **Anticipated starting date** |
| 01.06.20 |
| **Anticipated completion date** |
| Completion of rapid review: end August 2020 |
| **Contact** |
| Josefien Breedvelt  Centre for Urban Mental Health at the University of Amsterdam  Amsterdam UMC, location AMC, department of psychiatry and department of public health  <https://www.uva.nl/en/shared-content/zwaartepunten/en/urban-mental-health/urban-mental-health.html> |
| **Review team member and organizational affiliations** |
| Josefien Breedvelt, Centre for Urban Mental Health at the University of Amsterdam and department of psychiatry at the Amsterdam University Medical Centers  Dr. Iris Elliott, Irish Human Rights and Equality Commission, Dublin  Prof, Claudi Bockting, Centre for Urban Mental Health at the University of Amsterdam and department of psychiatry at the Amsterdam University Medical Centers  Dr. Claire Niedzwiedz, University of Glasgow  Evelyn Sharples, Independent Consultant  Prof. Sandro Galea, Boston University School of Public Health  Prof. Henning Tiemeier, Harvard TH Chan School of Public Health |
| **Funding sources** |
| Funding: Wellcome Trust |
| **Conflicts of interest** |
| None |
| **Collaborators** |
| Leaders Unlocked – an organisation promoting the voice of young people and under-represented groups (http://leaders-unlocked.org/).  Kleijnen Systematic Reviews (KSR) Ltd – an independent research company specialising in producing systematic review and analysis of health care evidence (https://www.systematic-reviews.com/). |
| **Review question / main purpose** |
| We have been tasked by Wellcome to provide an insight analysis on neighbourhood social cohesion.  The main question they aim for us to answer is ‘drawing inferences from the current evidence: in which ways and in which contexts and for whom does your chosen active ingredient appear to work, and why; and in which ways and in which contexts and for whom does it appear not to work, and why?’  We have decided to conduct a rapid review in response to this with the following review questions:   1. Which elements of neighbourhood social cohesion, and to what extent affect the mental health (depression and anxiety) of adolexents and young people (14 - 24 years) who are at risk of developing depression and/or anxiety? (etiological) 2. What is the effectiveness of interventions aiming to improve neighbourhood social cohesion for preventing depression and/or anxiety in adolescents and young people (14 - 24 years) at risk of developing depression and/or anxiety? (interventions) |
| **Searches** |
| An electronic search will be conducted in PubMed, and systematic review databases for peer review literature.  Searches will be conducted using search terms pertaining to children and young people, neighbourhood social cohesion and depression and anxiety.  Pubmed Search term:  ((((neighbourhood social cohesion) OR (social capital) OR (neighbourhood cohesion) OR (social cohesion) OR (community cohesion) OR (neighbourhood support) OR (neighbourhood networks)) AND ((prevention) OR (effects) OR (intervention) OR (public health)) AND ((youth) OR (young people) OR (children) OR (adolesc*) OR (students))) AND ((anxiety) OR (depression)))) Filters: Child: birth-18 years, Adolescent: 13-18 years, Young Adult: 19-24 years  1,597 – 24.06.20  An additional search using key search terms was conducted in KSR Evidence, a comprehensive database of systematic reviews in health care published since 2015, and Campbell Collaboration, conducted on 09.07.20.  Additional Grey literature databases were searched: OAIster, OpenGrey, Jisc Library Hub Discover, DH-DATA: Health Administration, Medical Toxicology & Environmental Health (Dialog), and Google. Additional mental health and social care websites were searched: Young Minds, Centre for Mental Health, Institute of Mental Health, Joseph Rowntree Foundation, and WHO Mental Health. All searches were conducted between 08.07.20 and 10.07.20.  All grey literature databases were searched with a combination of search terms encompassing the following:  ("neighbourhood cohesion" OR "neighborhood cohesion" OR "social cohesion" OR "community cohesion" OR "community participation" OR "community involvement" OR "community engagement" OR "social capital" OR "social network*") kw:("mental health" OR "mental disorder*" OR "mental illness*" OR depression OR depressive OR anxiety OR anxieties OR resilience) kw:("young people" OR "young adult*" OR youth OR child OR children OR adolescen* OR teenage* OR "emergent adult*" or "early adult*") |
| **Condition or domain being studied** |
| Our active ingredient is neighbourhood social cohesion, referring to “the extent of connectedness and solidarity among neighbours in society”^1^. This is characterised by the absence of social conflict (i.e. polarisation) and the presence of strong social bonds (i.e. trust, reciprocity). Factors that indirectly impact social cohesion will also be included such as built environment and neighbourhood composition. |
| **Participants/population** |
| Young people aged 14 - 24 at risk of developing depression or anxiety.  Inclusion:  Study population   - Studies reporting on outcomes in children and young people with the age range or mean age between 14 – 24 years - Within scope are young people who are at risk of depression of anxiety (assessed using a validated tool). This includes young people who do not meet diagnostic criteria for anxiety and depression, people with levels of symptomatology that meet mild to severe anxiety and depression as measured, and self-reported or identified experiences of anxiety and depression. This includes thoughts, feelings and behaviour as part of anxiety and depression that impair functioning or hold people back for a longer period (i.e. weeks).   Exclusion:  Study population   - If the study reports outcomes for young people above age 24 or below 14 - Studies focussing on the effect of neighbourhood social cohesion on various levels of stress, wellbeing, resilience, emotions thoughts and behaviours in response to daily events or substantial life events (i.e. death of family members) or symptoms related to severe mental health conditions including bipolar disorder and schizophrenia. We also exclude studies focussing on externalising conditions such as addictions or studies where young people have a primary diagnosis of a long-term health condition (i.e. diabetes, HIV) or studies where the primary outcome is suicidal ideation or suicide risk. |
| **Interventions/exposures** |
| Inclusion:  Intervention / exposure characteristics:   - Studies that aim to explore the effect or impact of neighbourhood social cohesion, social capital or community cohesion or specific factors related to cohesion in the community (specifically community peer relationships or community violence) and how it relates to mental health   Exclusion:  Primary topic of study on the following, unless also including an explicit focus on social cohesion within neighbourhoods:   - Interventions or exposures do not primarily affect community level cohesion (i.e. family level, individual level or global level exposures/interventions). - Interventions or exposures that are delivered in education settings institutions (schools, universities) - Interventions or exposures primarily pertaining to families (e.g. orphan, parental separation) or peer groups not specifically in a community or neighbourhood area |
| **Comparator(s)/control** |
| Where possible we search to report on studies with comparisons that allow us to infer causality:   - Migration study designs (change in neighbourhood and change in outcome) - Longitudinal cohort studies (preferably multicentre for generalisability) studies to infer core components of socially cohesive neighbourhoods - Quasi-experimental study designs (including studies with a control group - intervention vs no intervention, difference in difference study design etc) - Experimental study designs (randomised controlled trials)   Cross-sectional study designs will be included as well but only if we are unable to identify enough high-quality longitudinal and/or (quasi-experimental) study designs. |
| **Types of studies to be included** |
| Study characteristics:   - Systematic reviews, reviews, and meta-analyses - If these are not available, we will move to synthesise primary studies evaluating neighbourhood social cohesion either via (quasi)-experimental studies, longitudinal cohort study designs and finally cross-sectional studies. A hierarchy of evidence will be applied wherein we will prioritise inclusion of systematic reviews, (quasi) experimental study designs and longitudinal cohort study designs. - Published in English   Excluded   - Case reports, commentaries, editorials, protocols, books, book chapter, thesis, reviews (non-systematic) |
| **Context** |
| Absence of or different levels of neighbourhood social cohesion, social capital or community cohesion |
| **Main outcomes** |
| We focus on the prevention of anxiety and depression.   - Symptomatology or incidence of common mental disorders as assessed by validated (self-report) questionnaires/scales and/or interviews to ascertain onset of a mental health condition (e.g. PHQ-9, IDS, BDI, SCID, RCADS, GAD, SDQ, KIDSCREEN etc.) - We will exclude non-disorder specific outcome measures (e.g. wellbeing, resilience, loneliness, self-esteem), no full text available, language other than English or Dutch. |
| **Additional outcome(s)** |
| - Factors/elements affecting social cohesion - Interventions affecting social cohesion |
| **Data extraction** |
| Data will be extracted by Kleijnen Systematic Reviews with a cross check on 20% of studies conducted by a researcher at the Centre for Urban Mental Health.  A data extractor form will be developed in discussion with the research team. |
| **Risk of bias (quality) assessment** |
| The risk of bias (RoB) will be assessed in systematic reviews using the Risk of Bias Assessment Tool for Systematic Reviews (ROBIS) tool. The longitudinal studies will be assessed using the Joanne Briggs Institute (JBI) Critical Appraisal Checklist for Case Series. The cross-sectional studies will be assessed using the Joanne Briggs Institute Critical Checklist for Cross-sectional Studies. |
| **Analysis of subgroups or subsets** |
|  |
|  |
| **Type of review** |
| Systematic scoping review |
| **Health area of the review** |
| Prevention  Mental health  Children and young people |
| **Language** |
| English |
| **Country** |
| The Netherlands |
| **Dissemination plan** |
| The review will consist of a report formatted for submission to a scientific journal. The manuscript will include consideration of grey literature and lived experience accounts. Drafts will be reviewed by policy and lived experience colleagues to ensure accessibility.  In addition, we are working with an experienced scientific design & communication agency, to prepare the following materials for the funder:   - A 1-page lay narrative, providing a brief overview of what we found to be shared on a website aimed at general public. - A 1-page infographic summary, consisting of a visual overview aimed at relevant policy makers (e.g. public health government minister) explaining our results their implications for policy development. - A 2-minute video aimed at a 14-year old explaining the results and their implications for anxiety or depression in young people. |

1. **PICOS table**

**Inclusion/exclusion criteria:**

|  | **Include** | **Exclude** |
| --- | --- | --- |
| **P** | - Children and young people aged 14-24, including:   - Mean within this range   - Sub-group within this age range   - ‘young’ or ‘adolescents’ in grey literature - Young people who are at risk of depression of anxiety (assessed using validated tool), including:   - people who do not meet diagnostic criteria for anxiety and depression;   - people with levels of symptomatology that meet mild to severe anxiety and depression as measured   - self-reported or identified experiences of anxiety and depression (includes thoughts, feelings and behaviour as part of anxiety and depression that impair functioning or hold people back for a longer period (i.e. weeks)) | - - Young people >24 or <14 years of age   - Young people with a primary diagnosis of a long-term health condition (i.e. diabetes, HIV) or externalising conditions such as addictions   - Exclude non-disorder specific outcome measures (e.g., wellbeing, resilience, loneliness, self-esteem) |
| **I or exposure** | Neighbourhood social cohesion, social capital or community cohesion | Primary topic of study on the following, **unless** also including an explicit focus on social cohesion within neighbourhoods. Interventions or exposures that:   - do not primarily affect community level cohesion (i.e. family level, individual level or global level exposures/interventions). - Are delivered in education settings institutions (schools, universities) - primarily pertaining to families (e.g., orphan, parental separation) or peer groups not specifically in a community or neighbourhood area |
| **C** | Absence of or different levels of neighbourhood social cohesion, social capital or community cohesion |  |
| **O** | - Impact on mental health (anxiety and/or depression) - Factors/elements affecting social cohesion - Interventions affecting social cohesion - Barriers & facilitators of implementation of interventions | - The primary outcome is suicidal ideation or suicide risk - The effect on various levels of stress, wellbeing, resilience, emotions thoughts and behaviours in response to daily events or substantial life events (i.e. death of family member) or symptoms related to severe mental health conditions including bipolar disorder and schizophrenia |
| **S** | 1. Systematic reviews, and meta-analyses 2. If these are not available, primary studies evaluating neighbourhood social cohesion either via randomised controlled trials, longitudinal cohort study designs or quasi-experimental studies. | Case reports, commentaries, editorials, protocols, books, book chapter, thesis, reviews (non-systematic) |
| **Other** | Published in English |  |

1. **Lived experience workshop outline**

**Centre for Urban Mental Health & Leaders Unlocked**

**Involving young people in an insight analysis of neighbourhood social cohesion**

**Tuesday 23^rd^ June 2020: 2-4pm**

| ACTIVITY | RESOURCES |
| --- | --- |
| Welcome & Introduction   - Welcome and thanks for attending - Ground rules of virtual workshop - Brief introduction to organisations involved and young people - Agenda for the workshop - Any questions |  |
| Overview of project   - Josefien & Kristin to give a brief description / timeline of the project - Including why young peoples’ insight is so important |  |
| Pre-task   - Leaders Unlocked to invite each young person to share their pre-task. - *Why and how does your object/piece reflect your neighbourhood?* - *What are important elements of your local community?* |  |
| Exploring Social Cohesion   - Explore the groups’ definition of social cohesion and how they see the link with mental health.   Introduction:   - *What is neighbourhood social cohesion? **   (note down definitions and share on screen)  Exploratory conversation:   - *What do you see as the link between social cohesion and mental health?* - *Do you think social cohesion/ connection can help prevent anxiety/ depression in young people?*   Conclusion:   - *What are the most important elements of the local community that you see as beneficial for supporting mental health / preventing poor mental health?* - (Create a list, reflect as group and prioritise top 5) | Questions on slides  * Jim lives in a community which he feels has good social-cohesion. What might this look like?  What would be the signifiers for good social cohesion? |
| Literature Task and Thanks   - Introducing the selected literature and ask young people to each review a couple over the next week. - Thank you for attending, reminder next workshop Tuesday 30^th^ June - Any questions | UMH to provide selected literature on slides  (share screen) |

**Centre for Urban Mental Health & Leaders Unlocked**

**Involving young people in an insight analysis of neighbourhood social cohesion**

**Tuesday 30^th^ June 2020: 2-4pm**

| ACTIVITY | RESOURCES |
| --- | --- |
| Welcome & Recap   - Welcome and thanks for attending - Ground rules of virtual workshop - Brief recap of last session - Agenda for the workshop - Any questions |  |
| Initial Reflections   - Josefien to share brief reflections on last session |  |
| Pre-task   - Leaders Unlocked to invite each young person share their pre-task. - *Each person to share their example of good social cohesion either in their own area or elsewhere.* |  |
| Solutions for change   - Recap last weeks discussion on social cohesion/ key themes / top 5 priorities   Exploratory conversation:   - What needs to change in order to improve social cohesion? - How is a neighbourhood designed to facilitate this? - What type of interventions/solutions? - How can we translate our priorities into something that can affect change in the community? | See supporting questions below |
| Video style and design   - Josefien to explain why participants were asked to review the films, and our own plans - LU to invite YP to share their comments and reflections on the 4 short films |  |
| Next steps and thanks   - Next steps - Any questions |  |

Supporting Questions:

- *How do we make neighbourhoods safe e.g., green spaces?*
- *What makes a neighbourhood feel like you can talk to your neighbours?*
- *Is religion a key neighbourhood intervention?*
- *What is the perfect neighbourhood? How do you design the space and then support the people living there?*
- *Does there need to be a common goal?*
- *What about online communities – are you in touch with other people who live close to you?*
- *What type of approaches will help social-cohesion?*
- *A lot of the evidence is American-based (a lot about violence) What makes a neighbourhood safe?*

1. **PRISMA-P flowchart**

Excluded titles & abstracts: n = 1829

Full-text articles excluded: n = 389

Studies included:

n = 42

Systematic review n = 2

Longitudinal studies n = 13

Cross-sectional studies n = 27

Full-text articles assessed for eligibility: n = 431

Records screened: n = 2261

Records after deduplication: n = 2261

Additional records identified through other sources: n = 113

Records identified through database searching: n = 2302

1. **Baseline characteristics of cross-sectional studies and systematic reviews**

| 1. **Reference** | **Source of data** | **Study location;**  **type of community/ neighbourhood** | **Period of data collection or follow-up** | **Sample description (nr of participants; age [mean (SD, range)]; gender (%); ethnicity/race (%))** | **Outcome measure** |
| --- | --- | --- | --- | --- | --- |
| **Systematic reviews** | | | | | |
| **Hall 2018**{39} | **Included studies** (neighbourhood and community factors only):  Baams 2015  Burton 2013  Dahl 2009  Everett 2013  Fischer 2011  Gattis 2014  Heck 2011  Hightow-Weidman 2011  Kephart 2013  Khoury 2013  Rosario 2001  Thoma & Huebner 2013 | North America; city or NR | **Literature search:**  01/01/2000 - 10/07/2015 | **Baams 2015:** n= 876; 18.3 years (1.82); male 46%, female 54%; Latino 39%, White 20%, Black 25%, multiracial 23%, Asian 6%, American Indian 3%  **Burton 2013:** n=55; 17.0 years (1.36); male 15%, female 85%; person of colour 78%, White 22%  **Dahl 2009:** n=106; 20.1 years (1.8); male 29%, female 62%, 1% transsexual; White 75%, multiracial 10%, Latino 9%, Asian 4%, other 2%  **Everett 2013:** n=1328; 16.2 years (NR); male 28%, female 72%; White 75%, Latino 12%, Black 8%, Asian 3%, other 2%  **Fischer 2011:** n=1504; 16.3 years (1.3); male 39%, female 51%, 10% transgender; White 70%, Latino 10%, Black 6%  **Gattis 2014:** n=393; 23.4 years (5.3); male 33%, female 67%; White 75%, people of colour 25%  **Heck 2011:** n=145; 19.7 years (0.8); male 33%, female 60%, 7% transgender; White 71%, Black 10%, Asian 6%, Latino 5%, American Indian 1%, other 8%  **Hightow-Weidman 2011:** n=351; 20.4 years (2); male 100%; Black 68%, Latino 20%, multiracial 12%  **Kephart 2013:** n=52; 21.4 years (3.3); male 63%, female 37%; White 89%, multiracial 9%, Asian 2%  **Khoury 2013:** n=109; 21.3 years (2.2); male 36%, female 48%, 16% transgender; White 63%, multiracial 16%, Latino 8%, Asian 7%, Black 5%  **Rosario 2001:** n=156; 18.3 years (1.7); male 51%, female 49%; Latino 37%, Black 35%, White 22%, Asian or other 7%  **Thoma & Huebner 2013:** n=276; 17.5 years (1.4); male 59%, female 45%, 9% transgender; Latino 51%, White 49% | Child reported 20-item version of the BDI-Y (n=1 study), 21-item version of the BDI-II (n=3), 6-item version of the BSI (n=3), 10- or 20-item version of the CES-D (n=1 and 4, respectively) |
| **Perry 2015**{50} | **Included studies** (neighbourhood and community factors only):  DuRant 1995  Hammack 2004  Hawkins 1998  Hurd 2013  Nebbitt & Lombe 2008  Paxton 2004  Robinson 2011 | North America; NR, city or state | **Literature search:**  up to October 2014 | **DuRant 1995:** n=225; 14.4 years (2.2, 11 – 19 years); male 56%, female 44%; Black 100%  **Hammack 2004:** n=1704; median 15 years (13 – 18 years); male 45%, female 55%; African American 100%  **Hawkins 1998:** n=173; NR (13 – 15 years); male 39%, female 61%; African American 100%  **Hurd 2013:** n=571; 17.8 (0.65); male 48%, female 52%; African American 100%  **Nebbitt & Lombe 2008:** n=238; 15.6 years (2; 13 – 19 years); male 52.5%, female 47.5%; African American 100%  **Paxton 2004:** n=77; median 15 years (13 – 16 years); male 100%, female 0%; African American 100%  **Robinson 2011:** n=88; median 15 years; male 100%, female 0%; African American 100% | The CES-D (n=5), the CDI (n=1), the BSI (n=1). No information how many items or who reported symptoms. |
| **Cross-sectional studies** | | | | | |
| **Aneshensel 1996**{10} | NA | North America; province/county | October 1992 - April 1994 | **Overall:** n=877; 14.5 years (12 – 17 years); male 53.5%, female 46.5%; Latino 48.5%, non-Hispanic White 25.8%, African American 11.4%, Asian American 10.6%, other 3.6% | **Overall:** child reported 21-item version of the CDI; 8-item version of the Hopkins Symptom Checklist |
| **Behnke 2011**{59} | NA | North America; city | NA | **Overall:** n=383; 14.6 years (0.56; 14 – 16 years); male 47%, female 53%; Latino 100% | **Overall:** child reported 20-item version of the CES-D |
| **Black 2012**{57} | The Adolescent Mental Health, Behaviour and Life Experiences Study | Australasia; NR | May 2010 - August 2010 | **Overall:** n=520; 15.08 years (1.21; 13 – 18 years); male 44.3%, female 55.7%; NR | **Overall:** child reported 6-item KADS-6 |
| **Cheng 2014**{27} | The 2^nd^ phase of Well-Being of Adolescents in Vulnerable Environments (WAVE) study | North America, Asia  Africa; city | 2013 | **Baltimore:** n=456; 16.7 years (males and females, 15 – 19 years); male 57.7%, female 42.3%; NR  **New Delhi:** n=500; 16.6 and 16.7 years (males and females, respectively, 15 – 19 years); male 50%, female 50%; NR  **Ibadan:** n=449; 16.7 and 16.6 years (males and females, respectively, 15 – 19 years); male 46.9%, female 53.1%; NR  **Johannesburg:** n=496; 16.5 and 16.7 years (males and females, respectively, 15 – 19 years); male 54.8%, female 45.2%; NR  **Shanghai:** n=438; 16.8 and 16.9 years (males and females, respectively, 15 – 19 years); male 50.7%, female 49.3%; NR | **Overall:** child reported 10-item version of the CES-D |
| **Cooley-Quille 2001**{56} | NA | North America; rural | NA | **Phase 1:** n=185 (high-exposed n=89; low-exposed n=86); 15.43 years (1.27; 13 – 18; high-exposed 15.16 years (SD 1.12) and low-exposed 15.72 years (SD 1.39)); male 56.2% (high-exposed 46.5%; low-exposed 62.9%), female 43.8% (high-exposed 62.9%; low-exposed 37.1%)  **Phase 2:** n=33; NR; NR; NR; | **Phase 1:** child reported 20-item STAIC  **Phase 2:** child reported 27-item CDI; psychiatric interview using ADIS-C |
| **Delany-Brumsey 2014**{11} | The 1^st^ wave of the Los Angeles Family and Neighborhood Survey (L.A. FANS) | North America; province/county | April 2000 - January 2002 | **Overall:** n=564; 14.43 years (1.67, 12 – 17 years); NR; Latino 48.48%, White 25.96%, Asian/Pacific Islander 12.24%, Black/African American 12.11%, Native American 1.25% | **Overall: parent** reported 28 questions of the Behavioral Problem Index |
| **Duncan 2013**{1} | The 2008 Boston Youth Survey (BYS) Geospatial Dataset | North America; city | spring 2008 | **Overall:** n=1170; 16.31 years (1.27); male 44.2%, female 55.8%; White 10.33%, Black 42.47%, Hispanic 32.75%, Asian 7.36%, other 7.09% | **Overall:** child reported, adapted version of the MDS |
| **Dzhambov 2017**{34} | NA | Europe (non-UK); city | October - December 2016 | **Overall:** n=399; 17.89 years (2.27, 15 – 25 years); male 67.9%, female 32.1%; NR | **Overall:** child reported 12-item Bulgarian version of the GHQ-12 |
| **Elze 1999**{51} | The Youth Service Project | North America; city | NA | **Overall:** n=771; 15.3 years (13 – 18 years); male 43%, female 57%; African American (86%), other 14% | **Overall:** child reported DISC-R |
| **Epstein-Ngo 2013**{14} | NA | North America; city | NR | **Overall:** n=223; 14.5 years (0.69); male 39%, female 61%; Latino 100% | **Overall:** child reported 26-item version of the CDI |
| **Fitzpatrick 2005**{6} | NA | North America; province/county | spring 2002 | **Overall:** n=1538; 14 years (10 - 18 years); male 49%, female 51%; African American 100% | **Overall:** child reported 8-item version of the CES-D |
| **Lowe 2014**{36} | NA | North America; island | 2006/2007 | **All subpopulations:** n=1955; 15.3 years (0.95, 12 – 19 years); NR  **Jamaica:** n=278; 15 years (0.6, 14 – 16 years); male 41%, female 52%, unknown 7%; NR  **The Bahamas:** n=217; 14.3 years (0.9, 13 – 16 years); male 42%, female 52%, unknown 6%; NR  **St. Vincent:** n=716; 15.5 years (1, 14 – 18 years); male 46%, female 54%; NR  **St Kitts and Nevis:** n=737; 15.5 years (0.8, 13 – 17 years); male 48%, female 50%, unknown 2%; NR | **Overall:** child reported 21-item version the BDI |
| **Maurizi 2013**{17} | NA | North America; city | NR | **Overall:** n=202; 14.5 years (0.69); male 39%, female 61%; Latino 100% | **Overall:** child reported 26-item version the CDI (depression) and ‘’What I think and feel scale’’ (27-item revised version of the Children’s Manifest Anxiety Scale) |
| **McLaren 2015**{26} | NA | Australasia; state | NR | **Overall:** n=82; 16.54 years (1.05, 14 – 18 years); male 48.8%, female 51.2%; NR | **Overall:** child reported 20-item version the CES-D |
| **Mendelson 2010**{53} | NA | North America; city | September 2006 - February 2008 | **Overall:** n=677; 18.7 years (1.77, 16 – 23 years); male 50%, female 50%; African American 100% | **Overall:** child reported 20-item version of the CES-D |
| **Mitchell 2010**{12} | NA | North America; city | March 2005 - August 2007 | **Overall:** n=230; 22 years (1.5, 18 – 24 years); male 0%, female 100%; African American 100% | **Overall:** child reported 20-item version the CES-D |
| **Moses 1999**{58} | NA | North America; city | NA | **Overall:** n=337; NA (14 – 19 years); male 38%, female 62%; Black 44/5%, Hispanic 50.9%, Asian 0.9%, multi-ethnic origin 3.4% | **Overall:** child reported 12-item version of the SCL-90-R |
| **Nebbitt 2010**{40} | NA | North America; city | NR | **Overall:** n=238; 15.62 years (2.08, 13 – 19 years); male 52.5%, female 47.5%; African American 100% | **Overall:** child reported 20-item version the CES-D |
| **Pabayo 2016**{23} | The 2008 Boston Youth Survey | North America; city | spring 2008 | **Overall:** n=1641; NR (13 – 19 years); male 45.7%, female 54.3%; Black 41.6%, White 9.3%, Asian/South Asian 9%, Hispanic 33%, other 7.1% | **Overall:** child reported, adapted 5-item version of the MDS |
| **Rabbani 2018**{33} | NA | Asia; city | December 2016 | **Overall:** n=824; 23.6 years (3.6, 18 – 29 years); male 100%, female 0%; NR | **Overall:** child reported the GHQ-12 |
| **Rabinowitz 2016**{22} | NA | North America; NR | NR | **Overall:** n=631; 15.5 years (0.56, 14 – 16 years); male 72%, female 28%; White 76%, African American 22%, multiracial 2% | **Overall:** child reported 16-item the Mixed Anxiety and Depression subscale from the YSR |
| **Rose 2019**{2} | The National Survey of American Life-Adolescent (NSAL-A) | North America; NR | 2001-2003 | **All subpopulations:** n=1170; 14.98 years (1.41, 13 – 17 years); male 48%, female 52%; African American 93%, Caribbean Black 7%  **Unconnected:** n=135; 15.17 years (0.16); NR; African American 96%, Caribbean Black 4%  **Minimal connection:** n=436; 15.09 years (0.16); NR; African American 91%, Caribbean Black 9%  **High family connection:** n=50; 14.92 years (0.33); NR; African American 96%, Caribbean Black 5%  **Well-connected:** n=548; 14.86 years (0.09); NR; African American 94%, Caribbean Black 6% | **Overall:** child reported 12-item version of the CES-D |
| **Rosenthal 2001**{18} | NA | North America; borough | **College students:** 1994 - 1995  **Non-college students:** fall 1997 | **College students:** n=159; 18.4 years (16 – 20 years); male 25%, female 75%; Black or African-American 100%  **Non-college students:** n=92; 17.3 years (16 – 20 years); male 50%, female 50%; Black or African-American 100% | **Overall:** child reported multiple-item quasi-interval scales (general anxiety and depression) from the TSI |
| **Shukla 2015**{9} | The Understanding Health-Risk Behaviours Among Contemporary Adolescents (UHRBA) research project | North America; province/county | 2009 - 2010 | **Overall:** n=233; 17.01 years (0.62); male 39.5%, female 60.5%; African American 33%, Hispanic 54.9%, other 12% | **Overall:** child reported 6-item version of the MDD |
| **Stevenson 1998**{52} | NA | North America; city | NA | **Overall:** n=160; 14.6 years; male 45.6%, female 54.4%; African American 100% | **Overall:** child reported 47-item version of the SDMI |
| **Vilhjalmsdottir 2016**{24} | The Youth in Iceland (Yil) program | Europe (non-UK); country | March 2006 | **Overall:** n=5958; NR (15 – 16 years); male 49%, female 51%; NR | **Overall:** child reported 12-item version of the SCL-90 |
| **Zinzow 2009**{30} | The 2005 National Survey of Adolescents (NSA)-Replication study | North America; country | 2005 | **Overall:** n=3084; 14.6 years (1.7, 12 – 17 years); male 50.1%, female 49.9%; NR | **Overall:** the Depression Module of the NSA survey |

Abbreviations: ADIS-C - the Anxiety Disorders Interview Schedule for Children; BDI-Y – the Beck Depression Inventory for Youth; BDI-II – the Beck Depression Inventory-II; BSI - Brief Symptom Inventory; CDI - The Children's Depression Inventory; CES-D – the Center for Epidemiologic Studies Depression Scale; DISC – the Depression Intensity Scale Circles; KADS-6 – 6-item Kutcher Adolescent Depression Scale; MDD – the Major Depressive Disorder; MDS – the Modified Depression Scale; NA – not applicable; NR – not reported; SCL-90 - The Symptom Checklist-90; SDMI - short form of Multiscore Depression Index; S-MFQ - The Short Moods and Feelings Questionnaire; STAIC - the State-Trait Anxiety Inventory for Children; TSI – the Trauma Symptom Inventory; YSR -the Youth Self-report Inventory

**6a. Results of cross-sectional studies and systematic reviews**

| **Reference** | **Neighbourhood social cohesion factor** | **Social cohesion measure and/or theoretical framework** | **Results** |
| --- | --- | --- | --- |
| **Systematic reviews** | | | |
| **Hall, 2018**(4) | **Baams 2015:** feeling of thwarted social belonging (cross-sectional)  **Dahl 2009:** Positive and negative communal religious experiences (unpublished dissertation –  cross-sectional)  **Fischer 2011**: school belonging, community supportiveness of LGBQ people (unpublished dissertation – cross-sectional)  **Heck 2011:** community climate for LGBQ youth (cross-sectional)  **Kephart 2013:** interpersonal support, diversity of social network, social network contact (unpublished dissertation – cross sectional)  **Khoury 2013:** social support from friends (unpublished dissertation – cross-sectional) | No information was provided. | The feeling of thwarted belonginess was a risk factor (**Baams 2015**; *p*<0.05) and school belonging a protective factor (**Fischer 2011**; *p*<0.05) for the development of depressive symptoms. Positive or negative communal religious experiences, rejection by religious community (**Dahl 2009**; both *p*>0.05), community climate for LGBQ youth (**Heck 2011**; *p*>0.05) or length of association with an LGBQ student group (**Kephart 2013**; *p*>0.05) were not significantly correlated with depression. Social support from friends during childhood and adolescence or during emerging adulthood (**Khoury 2013**; *p*<0.05) or interpersonal support (**Kephart 2013**; *p*<0.05) were protective factors from depressive symptoms. Community support specifically for LGBQ was not shown to have significant protective effect (**Fischer 2011**; *p*>0.05). Conflicting results exist for correlation between social network contact and diversity of social network with depression (**Kephart 2013**). |
|  | **Baams 2015:** sexual orientation victimization  **Burton 2013:** sexual minority victimization (longitudinal)  **Dahl 2009:** Violence and harassment, religious-related sexual minority stress (unpublished dissertation –  cross-sectional)  **Fischer 2011:** victimization severity at school (unpublished dissertation – cross-sectional)  **Gattis 2014:** interpersonal discrimination (cross-sectional)  **Hightow-Weidman 2011**: racial and sexuality harassment (cross-sectional)  **Khoury 2013:** LGBQ victimization at school, teasing and bullying during childhood (unpublished dissertation – cross-sectional)  **Thoma & Huebner 2013:** experiencing racist or anti-LGBQ discrimination (cross-sectional) |  | Experiencing violence, victimization, harassment or, less often investigated, discrimination in community were risk factors significantly impacting the development of depressive symptoms (**Baams 2015, Burton 2013, Dahl 2009, Gattis 2014, Fischer 2011, Hightow-Wiedman 2011, Khoury 2013, Thoma & Huebner 2013**; all *p*<0.05). Social support from friends did not moderate the relation between teasing/bullying or victimization and depression (**Khoury 2013**; *p*>0.05). |
|  | **Everett 2013:** urbanicity of neighbourhood, proportion of registered republican voters in the area, proportion of college-educated residents in neighbourhood, proportion of same-sex couples in neighbourhood (longitudinal)  **Rosario 2001:** involvement in LGBQ activities (cross-sectional) |  | Urbanicity, but not change in neighbourhood urbanicity over time, was a significant risk factor correlated with depressive symptoms (**Everett 2013**; *p<*0.05 and >0.05, respectively). The decrease of republicans in the neighbourhood over the period 7 years, but not the proportion of republicans, was protective factor against depression (**Everett 2013**; *p*<0.05 and >0.05, respectively). The presence of college-education residents or same-sex couples in neighbourhood were not related to depressive symptoms (**Everett 2013**; *p>*0.05). There was no significant correlation between involvement in LGBQ nightlife and depression for both males and females (**Rosario 2001**; *p*>0.05). |
| **Perry et al., 2015**(5) | **DuRant 1995:** exposure to violence (cross-sectional)  **Hammack 2004:** neighbourhood exposure(cross-sectional)  **Hawkins 1998:** perceived future opportunities (cross-sectional)  **Hurd 2013:** neighbourhood characteristics  **Nebbitt & Lombe 2008:** exposure to high-risk neighbourhood, favourable attitude towards deviance (cross-sectional)  **Paxton 2004:** exposure to violence (cross-sectional)  **Robinson 2011:** exposure to violence (cross-sectional) | Two studies used the Survey of Exposure to Community Violence, and the Social Support Rating Scale (Paxton 2004; Robinson 2011). No more information was provided. | There was positive correlation between corporal punishment or exposure to neighbourhood violence and depression (r=0.32 and 0.28, respectively; *p*=NR; **DuRant 1995**). Exposure to violence was associated with significant impact on depressive symptoms in **Robinson 2011** (p=NR) and **Paxton 2004** (mean CES-D score 16.47, SD=9; a high risk for clinical depression).  **Hammack 2004** reported that exposure to neighbourhood had an impact on depression as 35% of males in the sample had the CES-D scores >16 indicating clinically significant depression.  The attendance at non-public schools had high impact on depressive symptoms as measured by CES-D (mean 21.8, SD=9.9; **Hawkins 1998**).  Living in neighbourhood with more African American residents (higher rates of residential stability) was associated with lower rates of depressive and anxiety symptomology. However, living in a neighbourhood with higher poverty and unemployment rates was associated with higher rates of depressive and anxiety symptomology (**Hurd 2013**).  **Nebbitt & Lombe 2008** reported that exposure to high-risk neighbourhood was associates with higher depression scores (CES-D; mean 20) in males when compared to mean in the sample (mean 28, SD 11.4). Favourable attitudes toward deviance was associated with depressive symptoms in male participants. |
| **Longitudinal studies** | | | |
| **Abada et al., 2007**(6) | perceived neighbourhood social cohesion (perceived safety, knowing and willingness to help neighbours, influence (role-model)) | **Perceived neighbourhood social cohesion:** the 6-item questionnaire measuring the extent to which the respondents perceives the neighbourhood to be safe, during daylight and after work, whether neighbours know and help each other, are friendly, and if there are adults that young people can look up to; reported by adolescents at cycle 4 | Neighbourhood characteristics (ethnic composition, median income, living in major cities and other large urban areas) did not have an impact on the development of depressive symptoms in cycle 4 (r^2^=0.157, *p*>0.05). The higher level of perceived neighbourhood cohesion is protective for the development of depressive symptoms at cycle 4 (r=-0.234, SE 0.091, *p*<0.05). |
| **Basáñez et al., 2013**(7) | neighbourhood composition | **Neighbourhood composition** information about the percentage of Hispanic living in the respondents' neighbourhood was determined by linking zip codes to US census 2000 data. | The effect of neighbourhood composition in 9^th^ grade did not impact the depression symptoms in 11^th^ grade (β=0.01, *p*=0.73). The impact of interaction of neighbourhood composition and discrimination on depressive symptoms was at borderline of significance (β=-0.07, *p*=0.06). |
| **Cerdá et al., 2011**(8) | violent victimization and witnessing violence | **Victimization**: subjects were asked whether they had been attacked with a weapon, beaten up, chased, shot at, sexually assaulted, or threatened with serious harm in the past year; reported at wave 2  **Witnessing violence**: questions adapted from ‘’My Exposure to Violence’’ (Selner-O’Hagan 1998) reported by subjects at wave 2 | Odds of depression at wave 3 following victimization and witnessing violence (past year) was 1.28 (95%CI 0.89-1.57, *p*=NR) and 1.27 (95%CI 0.81-2, *p*=NR), respectively. |
| **Donnelly et al., 2016**(9) | exposure to neighbourhood collective efficacy (social connections) | **Exposure to neighbourhood collective efficacy:** participants’ assessment using two adapted subscales derived from Morenoff (2001) | Higher neighbourhood collective efficacy (combined) seems to be protective from the development of depressive and anxiety symptoms in adolescents (age 15) (β=-0.114, SE 0.021, *p*<0.01 for both comparisons) even after controlling sociodemographic variables and previous mental health history (β=-0.073, SE 0.023, *p*<0.01 and β=-0.072, SE 0.023, *p*<0.01 for depressive and anxiety symptoms, respectively). |
|  | exposure to neighbourhood collective efficacy (perceived safety) |  |  |
| **Fredricks et al., 2006**(11) | extracullicular participation | **Extracullicular participation:** assessed at 11^th^ grade by participants; report on involvement in a range of activities such as school clubs (one question), organised sport involvement (two questions), volunteer service or civil rights activities (combined into prosocial activities; one question) | Participants taking part in sport activities showed significantly lower levels of depression as reported in 11^th^ grade (F(8,733)=10.03, np^2^=0.013, *p*<0.01), however, the impact was not significant for adolescents attending school clubs and prosocial activities (F=1.24, np^2^=0.002 and F=2.07, np^2^=0.000, respectively; *p*>0.05 for both). The impact of school clubs, sport activities or prosocial activities on the levels of depression was not significant at 1 year after high school (F=0.44, np^2^=0.001; F=2.26, np^2^=0.005; F=0.14, np^2^=0.0001, respectively, *p*>0.05 for all comparisons). The breadth of participation in 11^th^ grade or 1 year after high school was not associated with depression (β=-0.02 & -0.03, respectively, *p*>0.05 for both). |
| **Estrada-Martínez et al., 2012**(10) | exposure to neighbourhood stress (attitudes towards neighbourhood and neighbours, fear of violence) | **Attitudes** measured with 5-items and **fear of violence** with 2-items modified from Sampson and Wooldredge’s (1987) measure of social cohesion and trust reported by participants at Waves 5-8 | The correlation between the neighbourhood stress and the risk of developing depressive symptoms over time was significant (β=0.09, SE 0.02, *p*<0.001). |
| **Hurd et al., 2013**(12) | racial composition | **Racial composition:** assessed at the block group level with 2000 US census information | The neighbourhood racial composition was not predictive of participants depressive symptoms at wave 4 (t(109)=-0.75, *p=*0.455). Neighbourhood racial composition moderated the effect of higher levels of public regard (individuals’ perceptions of how other groups view their racial group) in predicting more symptoms of depression (t(109)=-2.44, *p*=0.016). |
| **Lee & Liechty, 2015**(13) | neighbourhood collective efficacy | **Neighbourhood collective efficacy:** five items reported by parents pertaining to social cohesion (3-items) and informal social control (2-items) | The neighbourhood collective efficacy (immigrant: OR 1.07 (95%CI 0.88-1.3, *p*>0.05) and non-immigrant: OR 0.94 (95%CI 0.75-1.17, *p*>0.05)) did not impact the odds of depression at wave 2. |
|  | perception of neighbourhood (safety and contentment) | Participants’ reported perceived **safety** (1-item) and perceived **contentment** (2-items) | Perceived neighbourhood contentment (immigrant: OR 1.06 (95%CI 0.75-1.5, *p*>0.05) and non-immigrant: OR 1.16 (95%CI 0.89-1.51, *p*>0.05)) or perceived neighbourhood safety (immigrant: OR 1.33 (95%CI 0.58-3.09, *p*>0.05) and non-immigrant: OR 0.82 (95%CI 0.37-1.77, *p*>0.05)) did not impact the odds of depression at wave 2. |
| **Solmi et al., 2017**(14) | Latino immigrant density | **Latino immigrant density** indicator was formed from three items in AddHealth’s contextual variables, based on the 19990 US Census  Set of 17 questions reported by mother during pregnancy, at 8 months postpartum and approximately at child ages two, three, five, seven, and ten years | The impact of Latino immigrant density on the odds of depression at wave 2 for immigrants and non-immigrants was 0.63 (95%CI 0.46-0.87, *p*<0.01) and 0.82 (95%CI 0.66-1.02, *p*>0.05). |
|  | neighbourhood social cohesion and trust |  | The odds of depressive symptoms at 18 years in neighbourhood with medium and low neighbourhood cohesion was 1.15 (95%CI 0.92-1.43, *p*>0.05) and 1.43 (95%CI 1.02-1.71, *p*<0.05), respectively. |
| **Viau et al., 2015**(15) | breadth, intensity and duration of participation in organised activities | Adolescents reported the number of different activity types (**breath of participation**), the average **intensity** of participation in activities and the combined number of years that they participate in activities (**the duration**) from Grade 8 to 11 | The duration of participation in organised activities at ages 14-17, but not breadth or intensity, was significantly correlated with lower depressive symptoms at age 18 (duration: β=-0.14, *p*<0.05; breadth: β=-0.07, *p>*0.05; intensity: β=-0.04, *p>*0.05). The social integration into peer group was an indirect mediator between the duration of participation and depressive symptoms (β=-0.02, SE 0.01, *p*<0.05) whereas support from activity leader did not mediate this correlation (β=-0.01, SE 0.01, *p>*0.05). The effect was significant for boys (β=-0.1, SE 0.03 *p=*0.002), but not girls (β=-0.01, SE 0.01 *p=*0.261). |
|  | support from the activity leader and social integration into the activity peer group | **Support from activity leader:** adapted 4-item scale from Mahoney et al. (2002) reported by adolescents  **Social integration:** adapted 5-item scale from Denault and Poulin (2008) reported by adolescents |  |
| **Wu et al., 2010**(16) | Community social capital (neighbourhood safety) | 5-item questionnaire regarding **community social capital** assessed by participants and their parents | Significant and negative correlation was reported for depressive symptoms and neighbourhood safety (r=-0.128, *p*<0.05). |
|  | Community social capital (neighbours concern and social capital) |  | Significant and negative correlation was reported for depressive symptoms and degree of neighbours caring about one’s life (r=-0.157, *p*<0.05). Higher levels of social capital were associated with lower level of depressive symptoms (β=-0.097, *p*<0.001). |
| **Cross-sectional studies** | | | |
| **Aneshensel and Sucoff, 1996**(17) | Social cohesion | 3 questions investigating if adolescents see themselves living in neighbourhoods where people are socially connected | Participants showed lower levels of depression, but not anxiety, with higher levels of neighbourhood social cohesion (β=-0.112, SE 0.032, p≤0.001 and β=0, SE 0.009, p>0.05, respectively). |
|  | Ambient hazards | **Ambient hazard** scale asked about 11 potential dangers (such as safety or violent crimes among others) assessed by adolescents | Participants showed higher levels of depression and anxiety with higher levels of neighbourhood ambient hazards (β=0.022, SE 0.008, p≤0.05 and β=0.009, SE 0.002, p≤0.001, respectively). |
| **Behnke et al., 2011**(18) | neighbourhood risk, societal discrimination | Youth perception of **neighbourhood risk** was assessed using a 12-item scale (10 risks and 2 protective factors reverse coded; Supple 2006); adolescents’ perception of **societal discrimination** was assessed using a 10-item scale (Whitbeck 2001) | The neighbourhood risk was associated with higher incidence of depressive symptoms in participants (β=0.38, *p<*0.05) with the total effect significant for both boys and girls (β=0.25 and 0.14, respectively; *p<*0.05 for both).  The societal discrimination was associated with higher incidence of depressive symptoms in participants (β=0.24, *p<*0.05) with the total effect significant for both boys and girls (β=0.1 and 0.24, respectively; *p<*0.05 for both). |
| **Black et al., 2012**(19) | socioeconomic status | **Socioeconomic status** was determined from the Australian Bureau of Statistics Socio-economic Index of Relative Socio-Economic Advantage and Disadvantage | The difference in socioeconomic status between participants screened as depressed and not depressed was at borderline of significance (mean=909.99 and 912.93, respectively; *p*=0.43). The difference was not significant for either females (mean=909.7 and 914.12, respectively; *p*=0.28) or males (mean=912.33 and 911.83, respectively; *p*=0.95). |
|  | degree of remoteness | **Degree of remoteness** was measured by home or school postcode using Accessibility/Remoteness Index of Australia plus (ARIA+) | The difference in ARIA+ scores between participants screened as depressed and not depressed where not significant for either males (median=3.83 and 4.15, respectively; *p*=0.25) or females (median=4.15 and 4.46, respectively; *p*=0.37). |
| **Cheng et al., 2014**(20) | perceived connection to the neighbourhood | **connection to the neighbourhood** was assessed with 3-items to identify whether adolescents perceived support on a community level | The adjusted odds of depressive symptoms as mediated through perceived connection to neighbourhood was significantly decreased only for males in Ibadan (OR=0.88, 95%CI 0.8-0.97, *p*<0.05), females in Johannesburg (OR=0.92, 95%CI 0.86-0.98, *p*<0.05), males and females in Shanghai (OR=0.72, 95%CI 0.55-0.93, *p*<0.05 and 0.75, 95%CI 0.71-0.79, *p*<0.05, respectively). The odds of depression for other comparisons ranged from 0.78 to 0.96 (*p*>0.05). |
| **Cooley-Quille et al., 2001**(21) | community violence | **Community violence** was assessed using the assessed Children’s Report of Exposure to Violence (32-item CREV; Cooley, Turner, & Beidel, 1995); adolescents were divided into low- (CREV score of <53) and high-exposure group (CREV score of ≥53) | Participants included in high-exposure group experienced more symptoms of trail anxiety (F(3,91)=3.6, *p*<0.05) than participant in low-exposure group. The difference was not significant between groups with regards to state anxiety (*p>*0.05) and depressive symptoms (*p*>0.05). Girls showed more anxious and depressive symptoms in high-exposure group when compared to girls in low-exposure group (t(44)=2.24, *p*<0.05).  Separation anxiety was positively correlated with total violence exposure (r=0.5, *p*<0.05) and witnessing violence (r=0.56, p<0.01). Depressive symptoms did not correlate with depressive symptoms (*p*>0.05). |
| **Delany-Brumsey et al., 2014**(22) | social capital | Randomly selected adults in the neighbourhood responded to 16 items developed by Sampson (1997, 1999) that assessed aspects of **social capital** | There was no association between social capital and internalising symptoms (β=0.51, SE=0.87, *p*>0.05). |
|  | residential stability (time not moving) and concentrated socioeconomic disadvantage | The indicator of **residential stability** was the percentage of people in the census track who reside in the same home in 1995 and in 2000; socioeconomic disadvantage was estimated from the Los Angeles Neighbourhood Services and Characteristics Database using 6 indicators | Living in more disadvantaged neighbourhood was associated with significantly more internalising symptoms (β=0.41, SE=0.21, *p<*0.05). There was no association between residential stability and internalising symptoms (β=-0.79, SE=1.55, *p*>0.05). |
| **Duncan et al., 2013**(23) | built environment | A range of **built environment** variables were characterised and were related to access to walking destinations (such as recreational open space density) and community design (such as sidewalk completeness). The youths’ neighbourhood was defined as 400- and 800-m street network buffers around the nearest intersection to their residence and created from StreetMap using the ArcGIS Network Analyst Extension | The Global Moran’s *I* value for depressive symptoms (*I=*0.092, *p*=0.034) indicated low positive spatial autocorrelation suggesting an impact on the depressive symptoms. The relationships between individual built environment features and depressive symptoms for the 400- and 800-m network buffers (all model 1) ranged from β=-0.741 to 0.418 (*p*=0.065 – 0.982). |
| **Dzhambov et al., 2017**(24) | social cohesion | Neighbourhood **social cohesion** was assessed by the brief form (9-item) of the Perceived Neighborhood Social Cohesion Questionnaire | Higher levels of social cohesion were associated with lower levels of mental health problems (β=-0.33, *p<*0.01). |
|  | restorative quality | Perceived **restorative quality** of the living environment was assessed with items from the Perceived Restorativeness Scale (PRS; Hartig 1997a, 1997b). | Higher levels of restorative quality were associated with lower levels of mental health problems (β=-0.18, *p<*0.01). Indirect paths linking road traffic noise and mental health (through social cohesion or restorative quality and social cohesion) showed statistically significant effect on participants’ mental health (β=0.04, 95%CI -0.0003-0.14, *p*=0.011 and β=0.01, 95%CI 0.0001-0.02, *p*=0.048, respectively). |
| **Elze et al., 1999**(25) | exposure to violence (victimization and witnessing violence) | **Neighbourhood and school violence** were assessed by adolescents with 2 (the extent to which shootings and murders occurred in their neighbourhood) and 2 items (shootings or knifings and teachers injured by students), respectively. **Extrafamilial personal victimization** was measured with one item from the DIS (whether youths were physically attacked or beaten in the last 6 months; Robins 1981).  **Witnessing a violent** act was measured by another item form the DIS (whether youths seen a person killed or seriously hurt). | The higher levels of personal victimization and witnessing a beating or murder were associated with higher levels of depressive symptoms (β=0.1 and 0.08, *p*<0.01 for both). The association was not significant for neighbourhood or school violence and depressive symptoms (β=-0.02 and 0.07, *p*>0.05 for both). |
| **Epstein-Ngo et al., 2013**(26) | exposure to violence (witnessing or being victimized) | Exposure to violence was measured with the Survey of Exposure to Community Violence, 20-item scale (Richters & Martinez, 1993). **Witnessing victimization** subscale consisted of 10-items investigating how often adolescents seen or directly witnessed various acts of community violence. **Personal victimization** subscale consisted of 10-items investigating how often adolescents had been directly victimizes by various acts of community violence. | The higher levels of victimization or witnessing violence were associated with greater levels of adolescents’ depression (β=0.23, *p*<0.001 and β=0.22, *p*<0.001). |
| **Fitzpatrick et al., 2005**(27) | social capital | **Social capital** was assessed by adolescents using 6 variables related to home, school and community. | The higher social capital index was correlated with the lower depressive symptoms (β=-0.07, *p*<0.05). |
|  | exposure to violence | **Exposure to violence** was assessed by adolescents and measured by two subscales: (1) a scale composed direct involvement with violence as a perpetrator (3 variables); (2) a scale that tapped aspects of adolescents’ victimization and their feelings about safety and threats in school, home and community (5 variables). | Exposure to violence as a victim was associated with higher levels of depressive symptoms (β=0.336, *p*<0.01). Exposure to violence as a perpetrator was not associated with changes in depressive symptoms (β=0.01, *p>*0.05). |
| **Lowe et al., 2014**(28) | social cohesion/support; the attachment to the neighbourhood | Adolescents’ perception of the community was measured using a modified, 38-item the Neighbourhood Characteristics Questionnaire (Barnes-McGuire 1997). The questions were modified to adolescents’ perspective. The questionnaire consisted of five subscales: (1) **social cohesion** (the extent to which people in the neighbourhood assisted and socialized with each other); (2) **the attachment to the neighbourhood** (the extent to which participants felt a part of their neighbourhood and their intentions of remaining in the neighbourhood); (3) **the neighbourhood quality** (the quality of life available in the neighbourhood and the extent to which the neighbourhood is in decline); (4) **the neighbourhood crime** (respondents’ perception of the safety of their neighbourhood, 9-items); (5) **the neighbourhood disorder** (the extent of organization in the community; items such as the presence of litter or public drinking). | Significant differences were found in adolescents’ perception of the social cohesiveness (F(3,1944)=17.32, *p*<0.01) and attachment (F(3,1944)=14.00, *p*<0.01) to the neighbourhood. Jamaican perceived their neighbourhood as more cohesive than other countries whereas residents of both, Jamaica and the Bahamas, were more attached to their neighbourhoods. Neighbourhood social cohesion was predictive factor of depressive symptoms only for residents of Jamaica (β=-0.13, *p*<0.05). Neighbourhood attachment was predictive factor of depressive symptoms for residents of Jamaica (β=-0.19, *p*<0.05) and St Kitts and Nevis (β=-0.22, *p*<0.05). |
|  | the neighbourhood quality and crime |  | Significant differences were found in adolescents’ perception of the quality (F(3,1944)=20.96, *p*<0.01) and crime (F(3,1944)=4.69, *p*<0.01) in the neighbourhood. St. Vincent perceived its neighbourhood as poorer than other countries whereas Jamaican perceived their neighbourhood to have lower levels of crime than other countries.  Neighbourhood quality was not a predictive factor of depressive symptoms only for any of the countries (all *p*>0.05). Neighbourhood crime was predictive factor of depressive symptoms only for residents of the Bahamas (β=0.2, *p*<0.05). |
|  | the neighbourhood disorder |  | Significant differences were found in adolescents’ perception of neighbourhood disorder (F(3,1944)=14.29, *p*<0.01) with Jamaican perceiving their neighbourhood as less disordered than other countries.  Neighbourhood disorder was predictive factor of depressive symptoms for residents of Jamaica (β=0.2, *p*<0.05) and St Kitts and Nevis (β=0.09, *p*<0.05). |
| **Maurizi et al., 2013**(29) | school belonging (through teacher and peer support, school activities) and neighbourhood belonging (through peer support and neighbourhood activities) | **School belonging** was assessed using 10 questions regarding adolescents’ sense of belonging (5 questions adapted by Goodenow (1993) from Bollen and Hoyle (1990); 4 items from Anderman’s (2003) and last item from Tyles and Degoey’s (1995)).  **Teacher support** was assessed using 8-item the Classroom Environment Scale (Moos & Trickett, 1987). **School peer support** was measured using 9-item trust subscale of the Inventory of Parent and Peer Attachment (Gullone & Robinson 2005).  **Neighbourhood belonging** was assessed using an adapted school belonging measured used in this study, but questions were tailored to neighbourhood instead of school. **Neighbourhood peer support** was assessed using the modified school peer support measure used in this study, but questions were tailored to neighbourhood instead of school.  **Involvement in school- and neighbourhood-based activities** was assessed by measure designed for the study; it reported how many times per week students engage in a variety of activities. | Teacher support and school peer support had statistically significant impact on school belonging (β=0.48 and 0.4, respectively; *p*<0.001 for both). Only neighbourhood peer support had statistically significant impact on neighbourhood belonging (β=0.3, *p*<0.001).  Higher level of school belonging was associated with lower levels of anxiety (r=-0.23, p<0.01; β=-0.19, *p*<0.01) and depression (r=-0.39, p<0.01; β=-0.39, *p*<0.001). Higher level of neighbourhood belonging was associated with lower levels of depression (r=-0.32, p<0.01; β=-0.16, *p*<0.05), but not anxiety (r=-0.12, *p*>0.05). |
| **McLaren et al., 2015**(30) | sense of belonging (through school, teacher and peer connectedness) | **Peer, school and teacher connectedness** were assessed by adolescents using 13-items from the Social Questionnaire for Secondary Students (Department of Education, Employment and Training, 2000). School and teacher connectedness scale consisted of five items whereas peer connectedness scale of four items.  **Sense of belonging** was assessed using the 18-item Psychological Subscale of the Sense of Belonging Instrument (Hagerty & Patusky 1995) modified to the gay, lesbian and bisexual youth group. | School belonging was significantly associated with school (β=0.36, *p*<0.001), teacher (β=0.46, *p*<0.001) and peer connectedness (β=0.42, *p*<0.001). School, peer and teacher connectedness were significantly associated with each other (β=0.37-0.56, *p*<0.001-0.01).  Higher levels of peer connectedness were associated with lower levels of depressive symptoms (β=-0.42, *p*<0.001); the results for school or teacher connectedness and depression were not statistically significant (β=0.12 and -0.14, respectively; *p*>0.05 for both). |
| **Mendelson et al., 2010**(31) | exposure to violence | **Exposure to violence** was assessed by adolescents using only three neighbourhood violence-related items taken from the Life Events Scale (D’Imperio, Dubow, & Ippolito, 2000). | The odds of high depressive symptoms (≥16) following exposure to neighbourhood violence were statistically significant (OR=2.27, 95%CI 1.57-3.3, *p*<0.001). The odds of high depressive symptoms remain significant if participants report single exposure variable - neighbourhood violence only (OR=2.29, 95%CI 1.5-3.5, *p*<0.001). |
| **Mitchell et al., 2010**(32) | violence exposure and ethnic discrimination | **Violence exposure** was assessed by mothers which completed the 27-item Survey of Exposure to Community Violence: Self-Report Version (Richters & Martinez, 1990) which measured how frequently they directly experiences or witnessed violence.  **Discrimination** was assessed by adolescents using the General Ethnic Identity Scale (Landrine 2006) measuring 17 specific discriminatory events and the extent to which the events were distressing. | There was a correlation between experiencing violence (r=0.37, *p*<0.001), witnessing violence (r=0.28, *p*<0.001) and ethnic discrimination (r=0.27, *p*<0.001) with depression. However, only higher levels of experiencing violence (β=0.32, SE=0.08, *p*<0.001) and ethnic discrimination (β=0.14, SE=0.06, *p*<0.05) were significantly associated with higher levels of depression. |
| **Moses, 1999**(33) | exposure to violence | **Exposure to violence** was measured by providing students with six violent events and asking how many times they had experiences each event. | For al participants, there was a positive association between violence against family or being raped and depression (r=0.112, *p*≤0.05 and r=0.212, *p*≤0.001 respectively). There was no correlation between depression and violence against friends (r=0.091, *p*>0.05), violence against strangers (r=0.007, *p*>0.05), being shot/stabbed (r=0.069, *p*>0.05) and being beaten up/jumped (r=0.024, *p*>0.05).  For males, the only positive and significant correlation was found for violence against family and depression (r=0.2, *p*≤0.05; all other r=0.04-0.15, *p*>0.05) whereas for girls between total exposure to violence and depression (r=0.14, *p*≤0.05; all other r=0.03-0.12, *p*>0.05).  The regression analysis showed the positive and significant association between exposure to violence and depression in females (β=0.15, *p=*0.031), but not males (β=0.058, *p=*0.522). |
| **Nebbitt & Lombe, 2010**(34) | perceived community cohesion and adultification | Neighborhood environment (**social cohesion** and **neighbourhood risk and disorganisation**) was assessed using the Subjective Neighborhood Scale that assesses a youth’s subjective appraisal of their neighbourhood (Aneshensel & Sucoff 1996). The scale consists of two relevant subscales: (1) 17-items assessing the level of perceived neighbourhood risk and disorganisation and (2) 3-items assessing perceived community cohesion.  **Exposure to delinquent peers** was assessed using the 14-item Exposure to Delinquent Peers Scale from the National Youth Survey (Elliot 1987) and report on how many of adolescent’s close friends are engaged in delinquent behaviours.  **Adultification**, the downward extension of adult responsibility to adolescents, was measured using 2 items. | Perceived community cohesion did not have statistically significant impact on depressive symptoms (r=-0.038, *p>*0.05; B=-0.415, SE=0.34, *p>*0.05). Adultification was associated with lower levels of depressive symptoms (r=-0.269, *p*<0.01; B=-1.016, SE=0.34, *p*<0.01). |
|  | exposure to delinquent peers, neighbourhood risk and disorganisation |  | Higher exposure to delinquent peers was associated with higher levels of depressive symptoms (r=0.385, *p*<0.01; B=0.32, SE=0.061, *p*<0.001). Higher exposure to neighbourhood risk was associated with higher levels of depressive symptoms (r=0.189, *p*<0.01; B=0.382, SE=0.142, *p*<0.01). |
| **Pabayo et al., 2016**(35) | Neighbourhood social cohesion/support | **Neighbourhood social cohesion** at neighbourhood level was reported by adolescents and measured using the Boston Neighbourhood Survey, adapting a previously used questionnaire (Sampson 1997). The survey consisted of five statements. Neighbourhood social cohesion at individual level was also measured using the same five statements. | There was no relationship between moderate or high neighbourhood-level social cohesion and depressive symptoms (moderate: β=0.12, 95%CI -0.02 to 0.26, *p*=NR; high: β=-0.06, 95%CI -0.24 to 0.12, *p*=NR) in contrast to individual-level moderate or high social cohesion (moderate: β=-0.16, 95%CI -0.28 to -0.05, *p*=NR; high: β=-0.26, 95%CI -0.4 to -0.13, *p*=NR). Social cohesion was not reported to be mediator between neighbourhood income inequality and depression. |
|  | income inequality, economic deprivation, danger, disorder | Census tract (CT)-level **income inequality** was the main exposure of interest, which was measured using the Gini coefficient (Kennedy 1996) for each census tract by the Boston Indicators Project.  **Economic deprivation**, a socioeconomic composite score, was created using US Census indicators including proportion of residents living below the poverty level, proportion of households receiving public assistance and proportion of families with a female head of household.  **Neighbourhood danger** was assessed using the data from the Boston Police Department. Counts of criminal homicide, robbery, aggravated assault, burglary, larceny theft, vehicle theft and arson were matched to the US Census Tracts.  **Disorder** scores were determined using data collected from the biennial, random-digit dial telephone survey, the Boston Neighbourhood Survey, and is comprised of combine score of social and physical disorders. | There was no relationship between income inequality and depressive symptoms (β=0.03, 95%CI -0.01 to 0.08, *p*=NR) for all participants, however, the analysis of females showed significantly higher depression scores (β=0.39, 95%CI 0.3 to 0.48, *p*=NR). Taking into account the level of census tract income inequality, the interaction was statistically significant (β=0.11, 95%CI 0.02 to 0.2, *p*=0.01).  Higher levels of economic deprivation were associated with lower levels of depressive symptoms (moderate: β=-0.13, 95%CI -0.27 to 0.01, *p*=NR; high: β=-0.18, 95%CI -0.32 to -0.04, *p*=NR). Moderate levels of danger were associated with higher levels of depressive symptoms whereas high levels of danger show no impact (moderate: β=0.18, 95%CI 0.04 to 0.32, *p*=NR; high: β=-0.03, 95%CI -0.14 to 0.09, *p*=NR). Moderate and high levels of disorder were not associated with change in depressive symptoms (moderate: β=-0.04, 95%CI -0.16 to 0.09, *p*=NR; high: β=-0.09, 95%CI -0.28 to 0.11, *p*=NR). |
| **Rabbani et al., 2018**(36) | social support | **Social support** was assessed by social network analysis showing direct friendship ties was performed following the respondents’ assessment of the number of close friend(s) in the community and the place of residency. | When compared to an isolated respondent (without friends), belonging to a small or large friendship group is associated with non-statistically significant decrease in depressive scores (better mental health; SD=-0.098, 95%CI -0.327 to 0.131, *p*>0.05 and SD=-0.117, 95%CI -0.274 to 0.014, *p*>0.05, respectively). Each additional friend in a friendship group is associated with respondents’ decrease in depressive score equal to SD=-0.063 (95%CI -0.106 to -0.021, *p*<0.01). |
| **Rabinowitz et al., 2016**(37) | neighbourhood social cohesion (sense of community, attraction-to-neighbourhood and social interaction within a neighbourhood) | **Neighborhood social cohesion** was assessed using the 18-items Neighborhood Cohesion Instrument (Buckner 1988) by adolescents. | Higher exposure to neighbourhood social cohesion was associated with lower levels of anxiety/depressive symptoms (r=-0.24, *p*<0.01; β=-0.21, *p*<0.01). The interaction between withdrawal and neighbourhood social cohesion predicted anxiety/depression symptoms (β=0.14, *p*<0.01) in contrast to sex, withdrawal and neighbourhood social cohesion interaction (β=0.05, *p*=0.247). |
|  | neighbourhood crime | **Neighbourhood crime** rates were otain by geocoding of the custodial parent address to the Pittsburgh Neighbourhood Maps (City of Pittsburgh GIS Division 2006) or TIGER 2000-based StreetMap USA data (ESRI 2002; US Census 2002). Total score was calculated by adding Part I and Part II crimes. | Neighbourhood crime was not associated with change in anxiety/depressive symptoms (r=-0.05, *p>*0.05; β=-0.03, *p>*0.05). The interaction between withdrawal and neighbourhood crime predicted anxiety/depression symptoms (β=0.11, *p*<0.05) in contrast to sex, withdrawal and neighbourhood crime interaction (β=0.02, *p*=0.735). |
| **Rose et al., 2019**(38) | neighbourhood connectedness (adolescents’ perceptions of their neighbourhood network) | **Neighbourhood** **connectedness** was measured using 4 items representing adolescents’ perception of their neighbourhood network adapted from the National Survey on Black Americans (Jackson and Neighbors 1997) and the National Longitudinal Study of Adolescent to Adult Health (Add Health). | Significant and negative correlation was reported for depressive symptoms and ‘neighbourhood’ social domain (r=-0.16, respectively; *p*<0.001). Subpopulation of ‘Unconnected’ participants (based on of social connectedness across family, peer, school, religion, and neighbourhood settings) reported higher levels of depressive symptoms (MD=11.37, SE=0.53) than participants in ‘Minimal connection’ (MD=9.54, SE=0.37), ‘High family connection’ (MD=9.89, SE=2) and ‘Well-connected’ (MD=7.9, SE=0.38) subpopulations (overall χ2 (3) = 35.64, p < 0.001). |
| **Rosenthal & Hutton, 2001**(39) | exposure to community violence (victimization and witnessing violence) | **Exposure to community violence** was collected using 18-items from the Survey of Exposure to Community Violence (Echters & Saltzman 1990) reported by adolescents. Seven and 11 items reflected the extent to which the individual had been a direct victim or witnessed community violence during high school, respectively. | There was no statistically significant correlation between victimization and anxiety (college vs non-college students: r=0.17 vs 0.27; *p*=NS for comparison) or depression (college vs non-college students: r=0.18 vs 0.31; *p*=NS). Similarly, no statistically significant correlation between witnessing violence and anxiety (college vs non-college students: r=0.17 vs 0.36; *p*=NS for comparison) or depression (college vs non-college students: r=0.11 vs 0.2; *p*=NS). There was no association between victimization and witnessing violence with either anxiety (college vs non-college students: r=0.2 vs 0.39; *p*=NS) or depressive symptoms (college vs non-college students: r=0.19 vs 0.32; *p*=NS). |
| **Shukla & Wiesner, 2015**(40) | exposure to school and neighbourhood violence (witnessing violence and violent victimization); neighbourhood hazard | **Violence exposure** was assessed by adolescents with two indicators: (1) witnessing violence and (2) violent victimization during the past year using 3 and 3-items form Youth Risk Behavior Surveillance Survey (Centers for Disease Control and Prevention, 2006), respectively.  **Neighbourhood hazards** were measured using 11-items from Aneshensel and Sucoff (1996) and reported by adolescents. | There was no association between witnessing violence in school or neighbourhood and depressive symptoms (β=0.12, SE=0.15, *p*>0.05 and β=0.03, SE=0.15, *p*>0.05, respectively). Similarly, no association was reported between victimization in school or neighbourhood and depressive symptoms (β=0.11, SE=0.2, *p*>0.05 and β=-0.04, SE=0.27, *p*>0.05, respectively). A statistically significant association was reported for neighbourhood hazards and depressive symptoms (β=0.2, SE=0.17, *p<*0.001). |
| **Stevenson, 1998**(41) | social capital, social support | **Neighbourhood social capital (NSC)** was measured using an 11-item scale that measures the degree a person perceives his or her neighbours to be aware and supportive of his or her activities and relationships (Stevenson 1997). Social capital was reported by adolescents.  **Social support** was measured using the 13-item Kinship Social Support (KSS) measure developed by Taylor (1993) as reported by adolescents. | There was significant and negative correlation between NSC and total SDMI in girls (r=-0.37, *p*<0.001), but not boys (r=0.02, *p*>0.05). There was no correlation between KSS and total SDMI (girls: r=0.03, *p*>0.05; boys: r=-0.01, p>0.05). Multiple regression analysis showed negative association between NSC and depressive symptoms (t=-2.16, *p*<0.05) even after controlling for gender and neighbourhood risk (t=-3.11, *p*<0.004). Similarly, living in high-risk neighbourhood with high level of social support was associated with lower levels of depressive symptoms (t=-2.52, *p*<0.02).  The total SDMI show that higher levels of NSC contribute to lower levels of global depression (low NSC mean=17.05, SE 8.1; high NSC mean=14.13, SE 6.3; *p*<0.05).  Positive and significant interaction was found between KSS and NSC (F(1,156)=3.73, *p*<0.05) with significant effects for adolescents with low levels of KSS (low KSS, low NSC: mean SDMI=17.26, SE 8.55; low KSS, high NSC: mean=13.75, SE 6.9), but now high levels of KSS (high KSS, low NSC: mean=16.12, SE 6.93; high KSS, high NSC: mean=15.29, SE 6.3). |
|  | calamity fear | **Calamity fear** was measured by the 6-item Fear of Calamity Scale developed by Stevenson (1997) and reported by adolescents. | There was no correlation between calamity fears and total SDMI (girls: r=-0.01, *p*>0.05; boys: r=-0.15, p>0.05). The multiple regression analysis showed that girls living in high-risk neighbourhoods and expressing high level of calamity fear tend to have lower depression scores (t=-1.89, *p*<0.06). |
| **Vilhjalmsdottir et al., 2016**(42) | social capital (parental social networks, neighbourhood reciprocity, neighbourhood contentment, sense of security, social trust) | **Social capital** at individual level, reported by adolescents, was measured with items referring to social ties among parents in the neighbourhood (Bernburg & Thorlindsson 2007; Coleman 1988), reciprocity between neighbours, contentment with the neighbourhood, feeling of security (Aminzadeh *et al.* 2013), collective efficacy (Sampson *et al.* 1997) and institutional trust as a social trust proxy (Lindstrom & Mohseni 2009; Mohseni & Lindstrom 2007). | Neighbourhood contentment (b=-0.15, SE=0.01, *p*≤0.001), sense of security (b=-0.06, SE=0.01, *p*≤0.001) and social trust (b=-0.1, SE=0.01, *p*≤0.001) are the only individual-level variables which showed a significant impact on participants emotional distress. |
|  | Income inequality | Neighbourhood **income inequality** was measured with the ratio of the mean disposable income of the top 20% highest income households to the mean disposable income of the 20% lowest-income households in the neighbourhood obtained from Statistics Iceland. | The income inequality has statistically significant impact on emotional distress after thoroughly controlling for individual and neighbourhood confounders as well as social capital (with social capital indicators: b=0.0034, SE=0.0015, *p*≤0.01; without: b=0.0025, SE=0.0017, *p*>0.05). |
| **Zinzow et al., 2009**(43) | exposure to community violence | **Violence exposure** was assessed using a module based on the Trauma Assessment for Adults and investigated 4 types of vents: serious accident, physical assault, sexual assault, natural disaster (Resnick 1996; Kilpatrick 2000).  **Witnessed community violence** was assessed using 6 questions. In addition, violence characteristics were based on the first violent incident that the adolescent witnessed and included multiples incidents witnessed, age on onset, age at last incident, location of the incident, relationship to the victim and fear of death or injury during the incident. | The odds of depressive episode following exposure to witnessed community violence were as follows: stabbing OR=1.56 (95%CI 0.98-2.48, *p>*0.05), sexual assault OR=2.11 (95%CI 1.01-4.42, *p<*0.05), mugging OR=1.69 (95%CI 1.06-2.7, *p<*0.05), threat with a weapon OR=1.66 (95%CI 1.09-2.54, *p<*0.05) and beating OR=2.01 (95%CI 1.36-2.97, *p<*0.001). The odds of major depressive disorder were significantly increased following multiple incidents of witnessed community violence (vs one incident; OR=2.18, 95%CI 1.44-3.31, *p<*0.001) and if participants were afraid that they might be injured or killed (vs no fear of injury or death; OR=1.9, 95%CI 1.22-2.98, *p<*0.01). |

Abbreviations: ARIA+ - Accessibility/Remoteness Index of Australia Plus; CREV - Children’s Report of Exposure to Violence; KSS - Kinship Social Support; MD - mean difference; NR – not reported; NS – not significant; NSC – neighbourhood social capital; RR – relative risk; SDMI – short form of Multiscore Depression Index; SE – standard error; US – the United States; 95%CI – 95% Confidence Interval

- 1. **Quality assessment**

**Risk of bias quality assessment for longitudinal studies (the Joanne Briggs Institute Critical Appraisal Checklist for Case Series(1))**

| **Study** | **Q1** | **Q2** | **Q3** | **Q4** | **Q5** | **Q6** | **Q7** | **Q8** | **Q9** | **Q10** |
| --- | --- | --- | --- | --- | --- | --- | --- | --- | --- | --- |
| **Abada 2007** | - | ? | + | - | ? | - | NA | + | - | + |
| **Basáñez 2013** | + | ? | + | ? | + | ? | NA | + | + | + |
| **Cerdá 2011** | ? | ? | + | ? | - | + | NA | + | + | + |
| **Donnelly 2016** | ? | ? | + | - | ? | + | NA | + | + | + |
| **Estrada-Martínez 2012** | - | ? | ? | ? | ? | ? | NA | + | ? | + |
| **Fredricks 2006** | - | + | + | ? | ? | ? | NA | + | ? | + |
| **Hurd 2013** | + | ? | + | ? | ? | ? | NA | + | ? | + |
| **Lee 2015** | ? | ? | + | ? | ? | ? | NA | + | ? | + |
| **Solmi 2017** | + | + | + | ? | + | + | NA | - | + | + |
| **Viau 2015** | + | ? | + | ? | ? | ? | NA | - | ? | + |
| **Wu 2010** | ? | ? | + | ? | + | + | NA | - | ? | + |

**Key:** Q1 - Were there clear criteria for inclusion in the case series? Q2 – Was the condition measured in a standard, reliable way for all participants included in the case series? Q3 – Were valid methods used for identification of the condition for all participants included in the case series? Q4 – Did the case series have consecutive inclusion of participants? Q5 – Did the case series have complete inclusion of participants? Q6 – Was there clear reporting of the demographics of the participants in the study? Q7 – Was there clear reporting of clinical information of the participants? Q8 – Were the outcomes or follow up results of cases clearly reported? Q9 – Was there clear reporting of the presenting site(s)/clinic(s) demographic information? Q10 -Was statistical analysis appropriate?

‘+’ indicates yes, ‘-’ indicates no, ‘?’ indicates unclear and 'NA' indicates not applicable.

**Risk of bias quality assessment for systematic reviews (A Risk of Bias Assessment Tool for Systematic Reviews (ROBIS)(2)**

| **Reference** | **Phase 2** | | | | **Phase 3** |
| --- | --- | --- | --- | --- | --- |
|  | 1. Study eligibility criteria | 2. Identification and selection of studies | 3. Data collection and study appraisal | 4. Synthesis and findings | **RISK OF BIAS IN THE REVIEW** |
| **Hall 2018** | ☺ | ☹ | ☹ | ☹ | ☹ |
| **Perry 2015** | ? | ☹ | ☹ | ☹ | ☹ |

Legend: ☺ = low risk; ☹ = high risk; ? = unclear risk

**Risk of bias quality assessment for cross-sectional studies (the Joanne Briggs Institute Critical Checklist for Cross-sectional Studies(3))**

| \| **Study** \| **Q1** \| **Q2** \| **Q3** \| **Q4** \| **Q5** \| **Q6** \| **Q7** \| **Q8** \| \| --- \| --- \| --- \| --- \| --- \| --- \| --- \| --- \| --- \| \| **Aneshensel 1996** \| ? \| + \| ? \| NA \| + \| + \| + \| + \| \| **Behnke 2011** \| - \| - \| + \| NA \| ? \| ? \| + \| ? \| \| **Black 2012** \| - \| - \| + \| NA \| ? \| ? \| + \| + \| \| **Cheng 2014** \| + \| ? \| ? \| NA \| + \| + \| + \| ? \| \| **Cooley-Quille 2001** \| - \| - \| + \| NA \| ? \| ? \| + \| ? \| \| **Delany-Brumsey 2014** \| ? \| ? \| ? \| NA \| + \| + \| + \| + \| \| **Duncan 2013** \| + \| + \| + \| NA \| + \| + \| + \| + \| \| **Dzhambov 2017** \| + \| ? \| + \| NA \| + \| + \| + \| + \| \| **Elze 1999** \| ? \| ? \| + \| NA \| + \| + \| + \| + \| \| **Epstein-Ngo 2013** \| ? \| ? \| ? \| NA \| + \| + \| + \| ? \| \| **Fitzpatrick 2005** \| ? \| ? \| ? \| NA \| + \| + \| + \| ? \| \| **Lowe 2014** \| ? \| - \| ? \| NA \| + \| + \| + \| + \| \| **Maurizi 2013** \| - \| ? \| + \| NA \| + \| + \| + \| + \| \| **McLaren 2015** \| - \| - \| + \| NA \| ? \| ? \| + \| + \| \| **Mendelson 2010** \| + \| ? \| + \| NA \| - \| - \| + \| ? \| \| **Mitchell 2010** \| + \| + \| + \| NA \| ? \| ? \| + \| + \| \| **Moses 1999** \| ? \| ? \| + \| NA \| + \| + \| + \| ? \| \| **Nebbitt 2010** \| - \| ? \| ? \| NA \| + \| + \| + \| + \| \| **Pabayo 2016** \| - \| ? \| ? \| NA \| + \| + \| + \| + \| \| **Rabbani 2018** \| + \| ? \| + \| NA \| + \| + \| + \| + \| \| **Rabinowitz 2016** \| + \| ? \| + \| NA \| + \| + \| + \| + \| \| **Rose 2019** \| + \| ? \| + \| NA \| + \| ? \| + \| + \| \| **Rosenthal 2001** \| - \| ? \| + \| NA \| ? \| ? \| + \| + \| \| **Shukla 2015** \| - \| ? \| + \| NA \| + \| + \| + \| + \| \| **Stevenson 1998** \| - \| ? \| + \| NA \| + \| + \| + \| + \| \| **Vilhjalmsdottir 2016** \| + \| - \| + \| NA \| + \| + \| + \| + \| \| **Zinzow 2009** \| - \| ? \| + \| NA \| + \| + \| + \| + \| |
| --- | --- | --- | --- | --- | --- | --- | --- | --- | --- | --- | --- | --- | --- | --- | --- | --- | --- | --- | --- | --- | --- | --- | --- | --- | --- | --- | --- | --- | --- | --- | --- | --- | --- | --- | --- | --- | --- | --- | --- | --- | --- | --- | --- | --- | --- | --- | --- | --- | --- | --- | --- | --- | --- | --- | --- | --- | --- | --- | --- | --- | --- | --- | --- | --- | --- | --- | --- | --- | --- | --- | --- | --- | --- | --- | --- | --- | --- | --- | --- | --- | --- | --- | --- | --- | --- | --- | --- | --- | --- | --- | --- | --- | --- | --- | --- | --- | --- | --- | --- | --- | --- | --- | --- | --- | --- | --- | --- | --- | --- | --- | --- | --- | --- | --- | --- | --- | --- | --- | --- | --- | --- | --- | --- | --- | --- | --- | --- | --- | --- | --- | --- | --- | --- | --- | --- | --- | --- | --- | --- | --- | --- | --- | --- | --- | --- | --- | --- | --- | --- | --- | --- | --- | --- | --- | --- | --- | --- | --- | --- | --- | --- | --- | --- | --- | --- | --- | --- | --- | --- | --- | --- | --- | --- | --- | --- | --- | --- | --- | --- | --- | --- | --- | --- | --- | --- | --- | --- | --- | --- | --- | --- | --- | --- | --- | --- | --- | --- | --- | --- | --- | --- | --- | --- | --- | --- | --- | --- | --- | --- | --- | --- | --- | --- | --- | --- | --- | --- | --- | --- | --- | --- | --- | --- | --- | --- | --- | --- | --- | --- | --- | --- | --- | --- | --- | --- | --- | --- | --- | --- | --- | --- | --- | --- | --- | --- | --- | --- | --- | --- | --- | --- | --- |
| **Key:** Q1 - Were the criteria for inclusion in the sample clearly defined? Q2 – Were the study subjects and the setting described in detail? Q3 – Was the exposure measured in a valid and reliable way? Q4 – Were objective, standard criteria used for measurement of the condition? Q5 – Were confounding factors identified? Q6 – Were strategies to deal with confounding factors stated? Q7 – Were the outcomes measured in a valid and reliable way? Q8 – Was appropriate statistical analysis used?  ‘+’ indicates yes, ‘-’ indicates no, ‘?’ indicates unclear and 'NA' indicates not applicable. |

- 1. **Lived experience workshop summary**

- 1. **Appendix references**

1. Joanna Briggs Institute. Critical Appraisal tools for use in JBI Systematic Reviews: Checklist for case series. Adelaide, Australia: JBI; 2020.

2. Whiting P, Savović J, Higgins JP, Caldwell DM, Reeves BC, Shea B, et al. ROBIS: A new tool to assess risk of bias in systematic reviews was developed. J Clin Epidemiol. 2016;69:225-34.

3. Joanna Briggs Institute. Critical Appraisal tools for use in JBI Systematic Reviews: Checklist for analytical cross sectional studies. Adelaide, Australia: JBI; 2020.

4. Hall WJ. Psychosocial risk and protective factors for depression among lesbian, gay, bisexual, and queer youth: A systematic review. J Homosex. 2018;65(3):263-316.

5. Perry DM, Tabb KM, Mendenhall R. Examining the effects of urban neighborhoods on the mental health of adolescent African American males: a qualitative systematic review. J Negro Educ. 2015;84(3):254-68.

6. Abada T, Hou F, Ram B. Racially mixed neighborhoods, perceived neighborhood social cohesion, and adolescent health in Canada. Soc Sci Med. 2007;65(10):2004-17.

7. Basáñez T, Unger JB, Soto D, Crano W, Baezconde-Garbanati L. Perceived discrimination as a risk factor for depressive symptoms and substance use among Hispanic adolescents in Los Angeles. Ethn Health. 2013;18(3):244-61.

8. Cerdá M, Tracy M, Sánchez BN, Galea S. Comorbidity among depression, conduct disorder, and drug use from adolescence to young adulthood: Examining the role of violence exposures. J Trauma Stress. 2011;24(6):651-9.

9. Donnelly L, McLanahan S, Brooks-Gunn J, Garfinkel I, Wagner BG, Jacobsen WC, et al. Cohesive neighborhoods where social expectations are shared may have positive impact on adolescent mental health. Health Aff. 2016;35(11):2083–91.

10. Estrada-Martínez LM, Caldwell CH, Bauermeister JA, Zimmerman MA. Stressors in multiple life-domains and the risk for externalizing and internalizing behaviors among African Americans during emerging adulthood. J Youth Adolesc. 2012;41(12):1600-12.

11. Fredricks JA, Eccles JS. Is extracurricular participation associated with beneficial outcomes? Concurrent and longitudinal relations. Dev Psychol. 2006;42(4):698-713.

12. Hurd NM, Sellers RM, Cogburn CD, Butler-Barnes ST, Zimmerman MA. Racial identity and depressive symptoms among Black emerging adults: The moderating effects of neighborhood racial composition. Dev Psychol. 2013;49(5):938-50.

13. Lee M-J, Liechty JM. Longitudinal associations between immigrant ethnic density, neighborhood processes, and Latino immigrant youth depression. J Immigr Minor Health. 2015;17(4):983-91.

14. Solmi F, Colman I, Weeks M, Lewis G, Kirkbride JB. Trajectories of neighborhood cohesion in childhood, and psychotic and depressive symptoms at age 13 and 18 years. J Am Acad Child Adolesc Psychiatry. 2017;56(7):570-7.

15. Viau A, Denault A-S, Poulin F. Organized activities during high school and adjustment one year post high school: Identifying social mediators. J Youth Adolesc. 2015;44(8):1638-51.

16. Wu Q, Xie B, Chou C-P, Palmer PH, Gallaher PE, Johnson CA. Understanding the effect of social capital on the depression of urban Chinese adolescents: An integrative framework. Am J Community Psychol. 2010;45(1-2):1-16.

17. Aneshensel CS, Sucoff CA. The neighborhood context of adolescent mental health. J Health Soc Behav. 1996;37(4):293-310.

18. Behnke AO, Plunkett SW, Sands T, Bámaca-Colbert MY. The relationship between Latino adolescents' perceptions of discrimination, neighborhood risk, and parenting on self-esteem and depressive symptoms. J Cross Cult Psychol. 2011;42(7):1179-97.

19. Black G, Roberts RM, Li-Leng T. Depression in rural adolescents: relationships with gender and availability of mental health services. Rural Remote Health. 2012;12:2092.

20. Cheng Y, Li X, Lou C, Sonenstein FL, Kalamar A, Jejeebhoy S, et al. The association between social support and mental health among vulnerable adolescents in five cities: findings from the study of the well-being of adolescents in vulnerable environments. J Adolesc Health. 2014;55(6 Suppl):S31-8.

21. Cooley-Quille M, Boyd RC, Frantz E, Walsh J. Emotional and behavioral impact of exposure to community violence in inner-city adolescents. J Clin Child Adolesc Psychol. 2001;30(2):199-206.

22. Delany-Brumsey A, Mays VM, Cochran SD. Does neighborhood social capital buffer the effects of maternal depression on adolescent behavior problems? Am J Community Psychol. 2014;53(3-4):275-85.

23. Duncan DT, Piras G, Dunn EC, Johnson RM, Melly SJ, Molnar BE. The built environment and depressive symptoms among urban youth: a spatial regression study. Spat Spatiotemporal Epidemiol. 2013;5:11–25.

24. Dzhambov AM, Markevych I, Tilov B, Arabadzhiev Z, Stoyanov D, Gatseva P, et al. Pathways linking residential noise and air pollution to mental ill-health in young adults. Environ Res. 2018;166:458-65.

25. Elze DE, Stiffman AR, Doré P. The association between types of violence exposure and youths' mental health problems. Int J Adolesc Med Health. 1999;11(3-4):221-5.

26. Epstein-Ngo Q, Maurizi LK, Bregman A, Ceballo R. In response to community violence: coping strategies and involuntary stress responses among Latino adolescents. Cultur Divers Ethnic Minor Psychol. 2013;19(1):38-49.

27. Fitzpatrick KM, Piko BF, Wright DR, LaGory M. Depressive symptomatology, exposure to violence, and the role of social capital among African American adolescents. Am J Orthopsychiatry. 2005;75(2):262-74.

28. Lowe GA, Lipps G, Gibson RC, Halliday S, Morris A, Clarke N, et al. Neighbourhood factors and depression among adolescents in four Caribbean countries. PloS one. 2014;9(4):e95538.

29. Maurizi LK, Ceballo R, Epstein-Ngo Q, Cortina KS. Does neighborhood belonging matter? Examining school and neighborhood belonging as protective factors for Latino adolescents. Am J Orthopsychiatry. 2013;83(2 Pt 3):323-34.

30. McLaren S, Schurmann J, Jenkins M. The relationships between sense of belonging to a community glb youth group; school, teacher, and peer connectedness; and depressive symptoms: testing of a path model. J Homosex. 2015;62(12):1688-702.

31. Mendelson T, Turner AK, Tandon SD. Violence exposure and depressive symptoms among adolescents and young adults disconnected from school and work. J Community Psychol. 2010;38(5):607-21.

32. Mitchell SJ, Lewin A, Horn IB, Valentine D, Sanders-Phillips K, Joseph JG. How does violence exposure affect the psychological health and parenting of young African-American mothers? Soc Sci Med. 2010;70(4):526-33.

33. Moses A. Exposure to violence, depression, and hostility in a sample of inner city high school youth. J Adolesc. 1999;22(1):21-32.

34. Nebbitt VE, Lombe M. Urban African American adolescents and adultification. Fam Soc. 2010;91(3):234-40.

35. Pabayo R, Dunn EC, Gilman SE, Kawachi I, Molnar BE. Income inequality within urban settings and depressive symptoms among adolescents. J Epidemiol Community Health. 2016;70(10):997-1003.

36. Rabbani A, Biju NR, Rizwan A, Sarker M. Social network analysis of psychological morbidity in an urban slum of Bangladesh: a cross-sectional study based on a community census. BMJ Open. 2018;8(7):e020180.

37. Rabinowitz JA, Drabick DAG, Reynolds MD. Youth withdrawal moderates the relationhips between neighborhood factors and internalizing symptoms in adolescence. J Youth Adolesc. 2016;45(3):427-39.

38. Rose T, McDonald A, Von Mach T, Witherspoon DP, Lambert S. Patterns of social connectedness and psychosocial wellbeing among African American and Caribbean Black adolescents. J Youth Adolesc. 2019;48(11):2271-91.

39. Rosenthal BS, Hutton EM. Exposure to community violence and trauma symptoms in late adolescence: comparison of a college sample and a noncollege community sample. Psychol Rep. 2001;88(2):367-74.

40. Shukla KD, Wiesner M. Direct and indirect violence exposure: relations to depression for economically disadvantaged ethnic minority mid-adolescents. Violence Vict. 2015;30(1):120-35.

41. Stevenson HC. Raising safe villages: cultural-ecological factors that influence the emotional adjustment of adolescents. J Black Psychol. 1998;24(1):44-59.

42. Vilhjalmsdottir A, Gardarsdottir RB, Bernburg JG, Sigfusdottir ID. Neighborhood income inequality, social capital and emotional distress among adolescents: a population-based study. J Adolesc. 2016;51:92-102.

43. Zinzow HM, Ruggiero KJ, Resnick H, Hanson R, Smith D, Saunders B, et al. Prevalence and mental health correlates of witnessed parental and community violence in a national sample of adolescents. J Child Psychol Psychiatry. 2009;50(4):441-50.
